# Bionic Perception of Surface Adhesion via a Magnetized Spring-like Sensor with Axial Stretchability

**DOI:** 10.1021/acsnano.5c07356

**Published:** 2025-06-22

**Authors:** Yuanzhe Liang, Biao Qi, Ming Lei, Yingyi Zhang, Yifan Liu, Yinning Zhou, Jianyi Luo, Bingpu Zhou

**Affiliations:** † Joint Key Laboratory of the Ministry of Education, Institute of Applied Physics and Materials Engineering, University of Macau, Avenida da Universidade, Taipa, Macau 999078, China; ‡ Research Center of Flexible Sensing Materials and Devices, School of Applied Physics and Materials, 47892 Wuyi University, Jiangmen 529020, China; § Department of Physics and Chemistry, Faculty of Science and Technology, University of Macau, Avenida da Universidade, Taipa, Macau 999078, China

**Keywords:** flexible tactile sensor, adhesion recognition, stickiness, 3D magnetized spring, laser processing

## Abstract

Perception of surface adhesion is one essential capability of a human fingertip, which is normally realized by touching the target surface with subsequent skin vibrations. However, such functionality is difficult to realize in flexible sensors and robotic systems due to the challenges in axial stretchability with reliable electrical feedback. In this study, we developed a bionic three-dimensional flexible magnetized spring (3D-FMS) that can quantitatively recognize surface adhesion based on electromagnetic induction. Combined with the laser processing with predefined patterns, we show that a raw flexible cube can be converted to highly stretchable spring-like geometry with excellent bidirectional deformation in axial orientation. Furthermore, the mechanical elongation caused by adhesion is critical for the induced voltage signals, allowing us to establish a model that relates adhesion strength with electrical outputs in a linear behavior. Via optimization of the process parameters, the device exhibits tailored stiffness to modulate the sensing sensitivity and working range on demand. With the established interactive interface, the wearable tests and robotic integration demonstrate the potential of the 3D-FMS for adhesion perception as a human fingertip. We expect that the strategy will offer a valuable reference to explore 3D wearable devices that advances robotic systems with more bionic functions such as stickiness determination.

## Graphical abstract

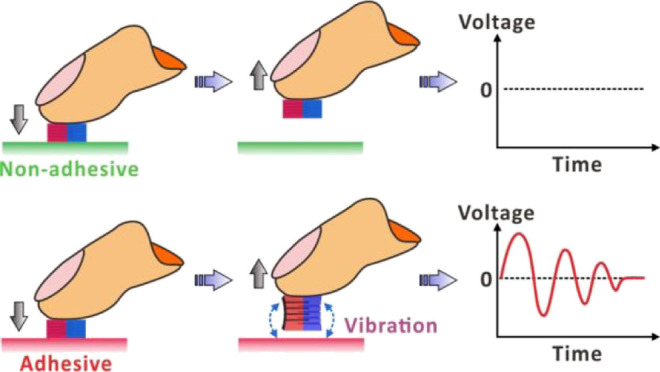

Today, robots are no longer just a tool for humans to perform simple and repetitive tasks in a number of specialized fields. The integration of disparate fields, including but not limited to computer science, graphology, materials science, and electronics,^1^ has led to a marked increase in the diversity and intelligence of robots. This development has had a profound impact on numerous industries and our daily lives. For instance, space robots have the capacity to perform a variety of tasks, including docking, berthing, repair, upgrading, rescue, and orbital debris removal;^2^ undersea robots can perform biological sampling^3^ and mineral exploration;^4^ and assistive robots offer the possibility of improving the lives of people with disabilities in mobility.^5^ In these scenarios, robots are often required to precisely grip materials like humans in intelligent and safe behavior. Many studies have been performed to achieve this goal. For instance, Wang et al. constructed a logarithmic spiral soft robot that can adapt to the shape of the target object for grasping like an octopus arm. The grasping size can span 2 orders of magnitude, and the grasping weight can exceed its own weight by 260 times.^6^ Boutry et al. developed an electronic skin that provides sensory feedback by mimicking the three-dimensional structure of the interlocking dermal–epidermal interface in human skin, providing a new solution for robot grasping to achieve slide detection and fragile object interaction.^7^ Despite the fact that robots are already very powerful in their grasping capabilities, it is still a challenge to sense and grasp some objects with special properties, e.g., objects with adhesive surfaces.

Adhesion is the tendency of different particles or surfaces preferring to cling to each other. As a physicochemical phenomenon, adhesion can be widely found in nature, as well as in human society, and plays an essential role. Geckos, for example, can easily walk on walls and ceilings by using the adhesion provided by the tiny setae densely packed on their feet;^8,9^ frogs prey with their remarkably sticky tongues;^10^ humans use a variety of glues and tapes to connect a wide range of items. Nowadays, adhesion has been widely applied to advance the fields of materials science,^11–13^ biomedicine,^14–16^ and aerospace.^17^ In short, the powerful functionality of human skin allows us to touch sticky surfaces without being affected by the subsequent creation of detached residues or excessive adhesion, which prevents facile detachment. But for robots, there is now the use of controlled surface adhesion to allow robots to manipulate objects in a nongrasping method.^18^ To date, few studies have been conducted on robots grasping objects with adhesive surfaces. To avoid accumulation of residue or manipulation failure during the robot grasping process, advanced diagnostic functions, e.g., adhesive perception, are of significant importance.^19^

Currently, the commonly used characterization methods of surface adhesion can be classified as micro- and macroscales. Atomic force microscopy (AFM) is one of the most popular methods in microscales,^20–22^ where the magnitude of adhesion force is obtained by special probes. However, the large dimension and complex operating procedures limit its practical applications in many cases.^23,24^ Recently, miniature adhesion sensors have been widely investigated because of the increasing call for device miniaturization and development of micronanoprocessing technology.^25,26^ However, the working range of these sensors is often with several thousand nanonewtons, which is relatively small for robotic applications. On the macroscale, surface adhesion measurements are still commonly performed using the traditional peel test or pull test,^27^ which are difficult for robotic integration.

Recent emergence of flexible sensors can be a good option for robots that offer high performance and good flexibility for tactile perception.^28,29^ Apart from the mechanical sensors, there are also a number of flexible sensors developed for viscosity,^30^ temperature,^31,32^ humidity,^33^ and gases.^34^ All of these flexible sensors have extended the robot’s ability to sense its surroundings for subsequent feedback responses. Owing to the structural limitation and mechanism of electrical feedback, however, perception of surface adhesion as a human being is still a challenge for flexible and wearable devices, which renders the concern of safe interaction with adhesive surfaces. Here, inspired by the daily operations, touch and release, of human fingertips, we reported a three-dimensional flexible magnetized spring (3D-FMS) for surface adhesion perception. Based on the application of unique laser cutting patterns, we show that a flexible spring-like device with tunable stiffness can be realized with excellent longitudinal stretchability. As a wearable device, the 3D-FMS could perform like the skins of a human fingertip to undergo a typical compression–elongation–rebound process. When in contact with an adhesive surface, the stretching finally results in spontaneous oscillation once it is detachable from the surface. Coupled with the built-in magnetized orientation, such mechanical deformation leads to rapid changes of magnetic flux to induce electrical signals in the coil layer, especially in the rebound phase. As the peak voltage is related to the degree of elongation, this allows us to associate the magnitude of surface adhesion with the electrical signals. We demonstrated that the sensitivity and working range can be flexibly regulated via sorts of parameters, e.g., the stiffness, to enable broad applications with specific requirement. Thanks to the compact size, the sensor could be equipped with a robotic arm to mimic the in situ adhesive perception with a fast and reliable response. We expect that the developed methodology and 3D-FMS prototype can potentially be an effective tool to promote the functionality of robots or artificial limbs for an intelligent future.

## Results and Discussion

### Design and Working Principles

When a human fingertip approaches an adhesive surface (e.g., a tape), we commonly cannot precisely feel the stickiness at the moment of touch. The sensation of stickiness/adhesion occurs mainly at the stage of finger detachment,^35^ which is accomplished by the cooperation of several organs (Figure 1a). As shown in Figure 1b, when our fingers touch and detach from the tape, the surface skins are continuously stretched before complete detachment. After being completely removed from the adhesive surface, the elongated finger skin vibrates and returns to its original state, which generates stimulation impulses that can be decoded by the nerve system, as depicted in Figure 1a. This allows the nervous system to decode the stimulation for stickiness recognition. From a bionic perspective, an adhesion sensor must possess several specific mechanical features, including compression, stretchability, and recovery. Spring-like geometry is natural to render these specific requirements. However, preparation of conventional springs requires the formation of a helical structure, which is challenging to realize without specialized equipment or methodology for flexible device production. Among the remarkable diversity of Chinese cuisine, the traditional food cutting technique, called “Basket Weaving Cut Technique”, is one approach to enable nondeformable solid foods with spring-like behaviors. As presented in Figure 1c, by creating intertwined cutting patterns on the original cucumber, the cucumber can simply be stretched to almost three times its original length in reversible behavior. In principle, the maximum stretching length can be easily adjusted by the cutting parameters such as space, depth, and angle. Inspired by this, we designed a three-dimensional flexible magnetized spring (3D-FMS), which is made through cutting on the two opposite faces of a cube followed by a magnetization process. Detailed fabrication processes of the 3D-FMS are shown in Figure S1, and the corresponding descriptions can be found in Methods. Figure 1d shows the overall layout of the 3D-FMS with total dimensions of 3 × 3 × 3.5 mm, where the stretchability and deformation were ensured after the application of laser cutting. Figure S2 provides the SEM images and EDS mapping results of NdFeB particles, the raw body, and the magnetized 3D-FMS. It can be observed that different elements were uniformly distributed within the 3D-FMS matrix (PDMS/Ecoflex). With embedded ferromagnetic NdFeB particles, the soft matrix can thus provide an alterable magnetic flux when exposed to mechanical deformations (Figure 1e). The equivalent spring model also shows the elasticity of magnetized dipoles can be positive to enable the change of localized magnetic flux during the stretching or compression process.

**Figure 1:**
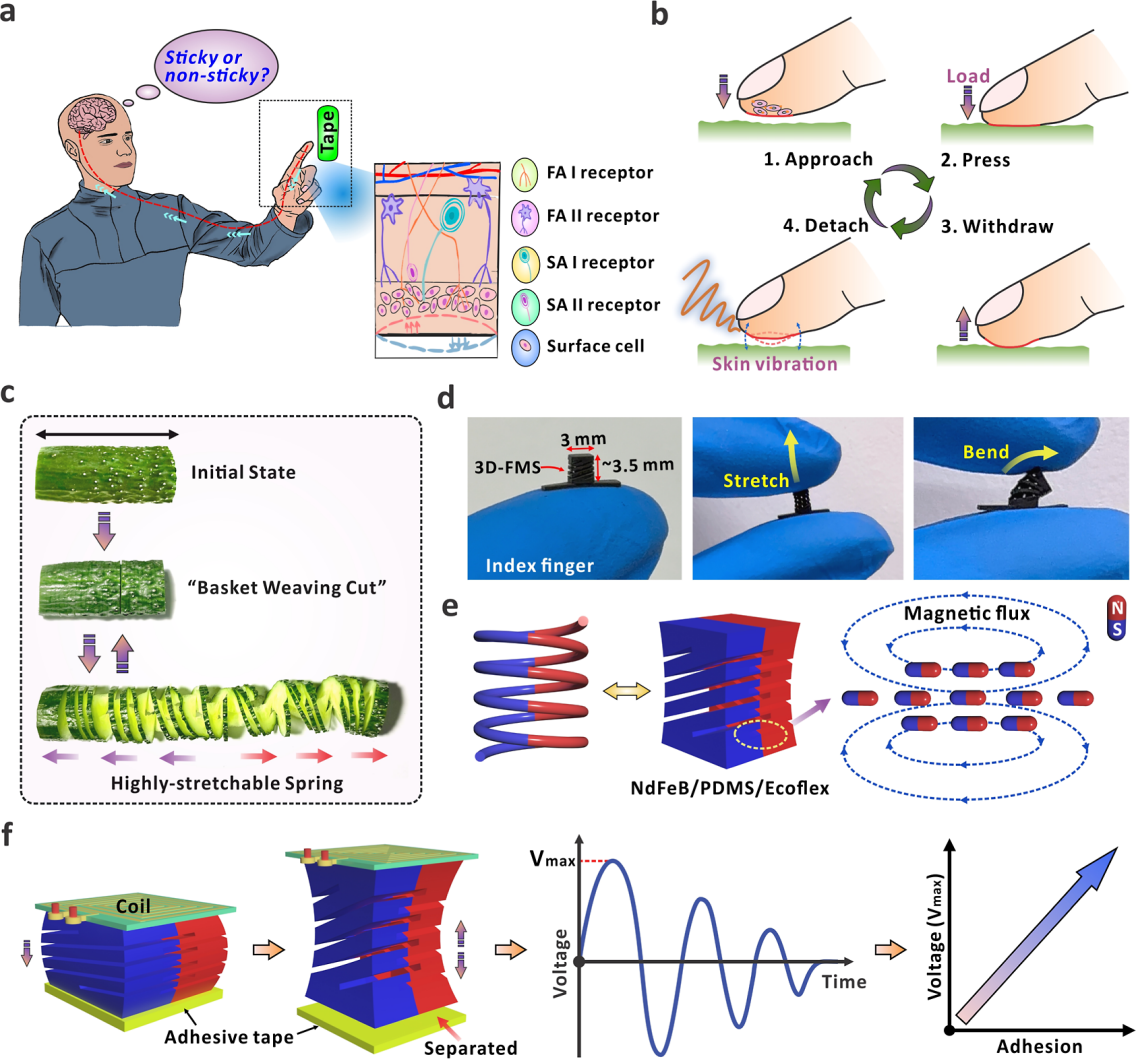
(a) Schematic diagram of how humans feel surface adhesion via touching a surface with signals transmitted through neural receptors. (b) Schematic diagram of the process when fingertip skins touch, press, and detach from an adhesive surface with vibration. (c) The traditional Chinese food cutting technique “Basket Weaving Cut Technique” for a stretchable cucumber. (d) Optical images of the miniature 3D-FMS with demonstration of flexibility and stretchability. (e) Diagrams of the spring model and the magnetic flux distribution of the magnetized 3D-FMS. (f) Schematic diagram of the sensing principle for surface adhesion based on the developed device.

The working mechanism for the 3D-FMS is shown in Figure 1f. When in contact with a tape via application of a corresponding normal force, the bottom face of the 3D-FMS would adhere onto the tape. Normally, the pressure would continuously lead to the compression of the 3D-FMS to ensure a tight adhesion with the tape. During the withdrawal of 3D-FMS from the tape surface, the spring-like structure would be first stretched, followed by an instant rebound to completely release from the surface. This is attributed to the continuous increase of inherent elastic force from the 3D-FMS, which finally exceeds the adhesion strength to render a necessary spring-like rebound. At this stage, the integration of a flexible copper coil layer (Figure S3) could perceive the spatial change in magnetic flux caused by the rapid rebound of 3D-FMS, which was then translated into an induced current governed by Faraday’s law of electromagnetic induction. A maximum voltage (*V*_max_) can then be extracted from the electrical profile with signal processing. Normally, a more significant stretching of the spring-like structure can be obtained if the device was touching the surface with larger interfacial adhesion. This behavior finally results in a higher electromotive force at the moment of separation due to the significant variation of magnetic flux and faster instantaneous rebound. Consequently, a quantitative relationship between the adhesion strength of a specific surface and the peak voltage can be established as shown in the schematic curve. From this perspective, the magnetized device with 3D architecture is essential to mimic the function of human fingertips for adhesion sensing, which is attributed to the reversible axial deformation (stretching/compression) with identifiable electrical feedback.

### Modeling and Optimization of 3D-FMS

As mentioned above, the maximum stretching displacement of 3D-FMS can be flexibly adjusted based on parameters such as cutting angle, depth, and space during the laser processing. Since the sensing performance is closely related to the stretching capability, we first investigated the effect of laser parameters on the tensile properties of the 3D-FMS. From this perspective, we consider that the mechanical property, e.g., the stiffness, of 3D-FMS can be analogous to a typical coil spring (Figure 2a). For cylindrical coil springs (e.g., axial compression/extension), the stiffness (*k*) can normally be described as “ $\frac{Gd^{4}}{8D^{3}N_{c}}$ ”, where *G* is the material modulus, *d* is the diameter of the spring wire, *N*_c_ is the effective coil number, and *D* is the average spring diameter.^36^ As presented in the figure, once the helix angle (α) is greater than 10°, the effective coil number *N*_c_ needs to be corrected as $N_{c}^{\prime }=\frac{H}{\pi D\cdot \tan\alpha }$ for a given height (*H*).^37^ Based on the above equivalent model, we then can regulate the mechanical property of the device using the key parameters, as depicted in Figure 2b. For a raw cube with side length (*L*) of the bottom surface and height of *H*, a typical spring-like structure can be formed after a standard laser processing with defined parameters. Herein, the “cut cycle” (*C*) represents the number of times each cut line is swept by the laser, “cut space” (*S*) is the distance between two adjacent cut lines in the axial (*z*) direction, and “cut angle” is the angle (θ) between the laser trajectory and the horizontal dimension. As discussed below, such parameters are critical to affect the stiffness, namely, stretching property, of the 3D-FMS. Here, we defined face 1 of the raw body as “cutting surface” and the neighboring face as “intersecting surface” (face 2). To systematically investigate the dependence of stiffness on the geometry of 3D-FMS, we customized the experimental setup, as shown in Figure S4. When exposed to an axial stretching force as shown in Figure 2c, the linear displacement of the device was recorded to reflect the stiffness as $k=\frac{F}{\delta }$ , where the applied force (*F*) was measured by the gauge and the displacement (δ) was recorded by the platform in real time. With different cut cycles on the same laser trajectory, the typical stretching behavior of the resultant 3D-FMS is presented in Figure 2d. For the raw cube, it can be observed that negligible displacement was obtained when a force of 0.05 N was applied to the device. Even though the original matrix is composed of flexible components, the unprocessed cube is difficult to exhibit axial stretching for adhesion determination. Obviously, the cutting depth on the intersecting surfaces increases if more cutting cycles were applied, which has been indicated by the yellow arrows in the optical images. This renders an increased overlapping region that is positive to enable a looser geometry for stiffness reduction (Figure S5). In principle, the increase in cutting depth brought about by more cycles will result in a larger average diameter of the 3D-FMS and thus the decrease in stiffness. Figure S6 also presents the stretching behavior when viewed from face 1 (cutting face), where a larger displacement exists by the 3D-FMS with 80 cutting cycles. However, once the number of cutting cycles is greater than 120, the cutting depth is saturated, and the variation is insignificant even when the value of “*C*” is further increased (Figure S7). Consequently, we optimized the cutting cycle from the perspective of sensor performance and processing convenience for the following investigations. Without further statement, the 3D-FMS device applied for subsequent characterizations and demonstrations is prepared by a cutting cycle of 80.

**Figure 2:**
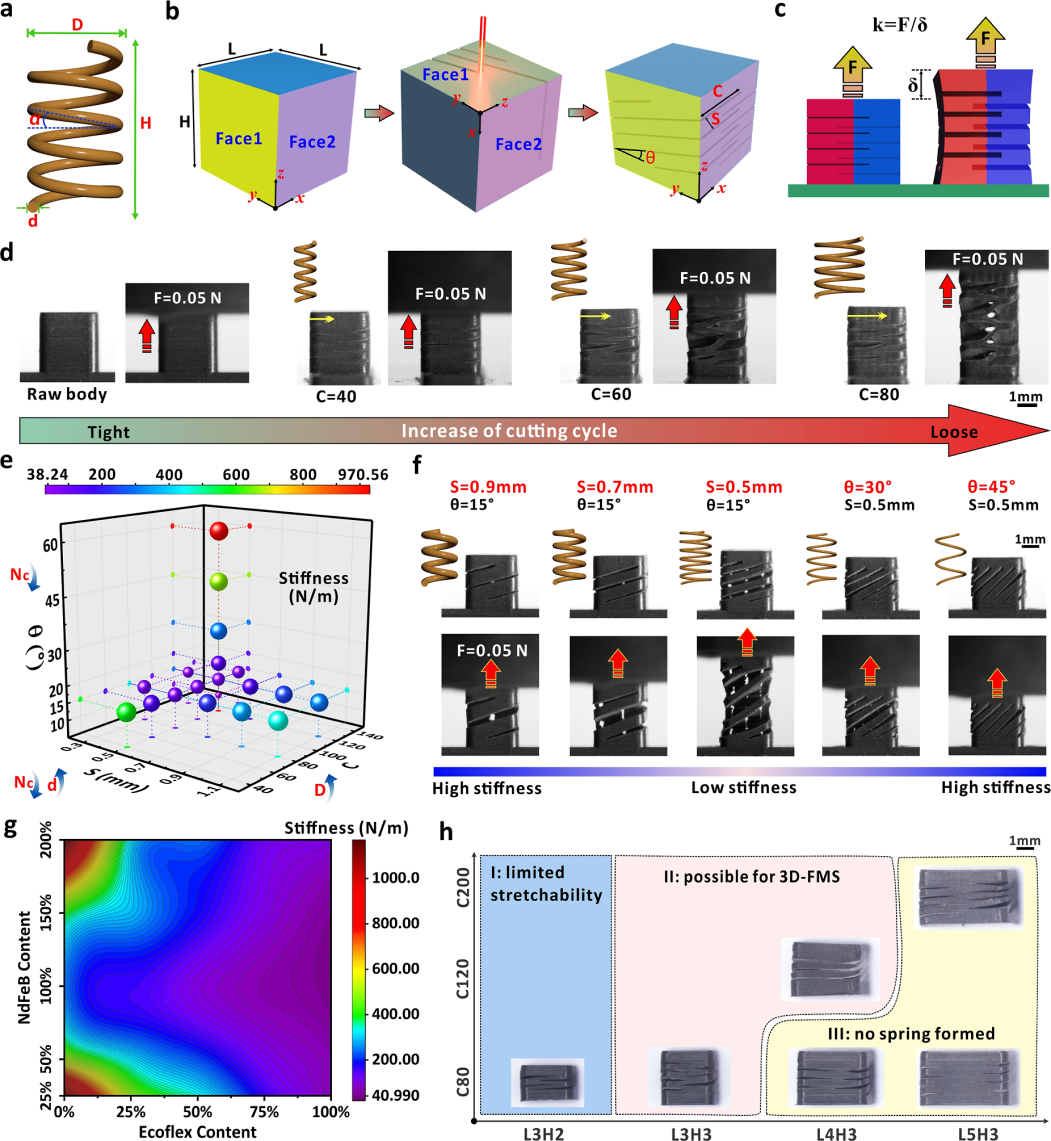
(a) Schematic diagram of the spring model with key parameters that affect the stretching/compressing performance. (b) Schematic diagram of the key parameters that play an important role in the 3D-FMS performance, including bottom side length (*L*), height (*H*), and cutting angle (θ), space (*S*), and cycle (*C*). (c) Schematic diagram of the experimental setup to measure the stiffness of 3D-FMS. (d) Stretching behavior of the 3D-FMS prepared by different cutting cycles. All samples were prepared via a space of 0.5 mm and angle of 15°. (e) Stiffness values based on different combinations of the cutting angle, space, and cycle during laser processing. (f) Optical images of the stretching behavior from different devices when the cutting space and angle were varied. The spring models were also provided, and all samples were prepared via a cycle of 80. (g) Stiffness of the laser-processed 3D-FMS based on different NdFeB and Ecoflex contents in the composite matrix. (h) Formation summary of the 3D-FMS based on different dimension of the raw body and cutting cycles. In the *y*-axis, *C* means cycle; in the *x*-axis, *L* and *H* indicate the length and height of the device.

Figure 2e further summarizes the stiffness of the 3D-FMS based on different combinations of cutting parameters (see Table S1 for detailed values). Some specific measurement curves, e.g., on different cutting angles, are provided in Figure S8 with detailed discussion. It can be observed that the stiffness of the 3D-FMS can be flexibly regulated based on the combinational effects from cutting angles, space, and cycles. Normally, the variations of cutting angle (θ), cycle (*C*), and space (*S*) are fundamental to regulating the key parameters of a spring model such as *N*_c_ (coil number), *d* (wire diameter), and *D* (spring diameter). The optical images in Figure 2f further present the stretching behavior of the devices when prepared by different parameters of space and angle. The equivalent spring models were provided alongside, which indicates the geometrical variation that determines the stiffness. An increase in the cutting space corresponds to a simultaneous reduction in the effective coil number and an increase in the diameter of the spring wire; thus, the stiffness of the 3D-FMS is increased as summarized in Figure 2e. When exposed to the same stretching force, the 3D-FMS with a smaller cutting space can then achieve a more obvious displacement. A detailed view of the cutting surface is provided in Figure S9, which presents that the width of the helix has been increased and the effective number of cut lines decreases if a larger space is applied during the laser processing. Note that if the cutting space on the raw body is relatively small, e.g., 0.3 mm, the excessive heat in a localized region would lead to burnout of the device (Figure S10). Furthermore, variation of the cutting angle mainly affects the helix angle of the spring and thus the effective helix number within the given height of the raw body. For example, the number of stretchable helices clearly decreases if θ was changed from 15° to 45°. As presented in the optical images, most of the helices were connected to the base of the device if a larger cut angle (60°) was applied. This limits the stretchability of the overall structure, which, in turn, increases the stiffness of the spring (as shown by the red dashed circles in Figure S11).

Apart from the morphological difference based on cutting parameters, we also measured the stiffness of the devices prepared by different mass ratios of the Ecoflex and NdFeB particles^38–40^ (see detailed stiffness values in Tables S2 and S3). As discussed below, the resultant stiffness variation will then provide a feasible solution to regulate the sensing performance, e.g., sensitivity or working range. For the raw bodies, the stiffness increases from a minimum of 100.22 to a maximum of 4456.8 N/m if the content of NdFeB particles increases and the ratio of Ecoflex in the elastic PDMS/Ecoflex matrix decreases (Figure S12). Such stiffness variation is mainly contributed by the elastic modulus that is highly dependent on the filling particles and compositions of the matrix.^41,42^ After laser processing, the 3D-FMS exhibits similar stiffness increases from 40.99 to 1171.1 N/m for the same mass ratios (Figure 2g). For the same material composition, however, obvious stiffness decay was observed after laser processing. The results experimentally confirm that the application of laser cutting has a significant contribution to reduce the stiffness of the device, which facilitates the stretching/compression behavior. Note that for the same Ecoflex content, a smaller NdFeB incorporation, e.g., 25% and 50%, exhibits a higher stiffness when compared with the higher NdFeB ratio (e.g., 100%). This is mainly attributed to the dependence of cutting depth on the transparency of related samples as shown in Figure S13. It can be observed that with a smaller NdFeB content, the cutting depth is reduced so that the resultant stiffness of 3D-FMS is inversely higher when compared with the devices with a larger NdFeB amount. In addition to the cutting parameters, the dimensions of the raw body also play an important role in affecting the formation yield of 3D-FMS. As depicted in Figure 2h, we mainly investigated the formation yield based on different lengths (*L*) of the bottom surface and the height (*H*) of the device. When the height is 2 mm, the actual processable height is 1 mm because we have defined the top and bottom 0.5 mm as the base. Consequently, the stretchability of the resultant device is limited even though the spring-like geometry can be successfully formed. With a height of 3 mm, the device of L3H3 (length of 3 mm and height of 3 mm for the raw body) is of potential for applications thanks to the spring-like formation with stretchability. Based on the “Basket Weaving Cut Technique”, the overlapping region in face 2 allows the overall device to easily stretch along the axial direction. With a further increased length to 4 mm (L4H3), it is difficult to ensure the sufficient overlapping region for stretching if the cutting cycle is 80. Only when the cutting cycle was further increased, we can observe that the spring-like geometry is formed as shown in the optical image (L4H3, C120). However, the overlapping region cannot be formed for the device of L5H3 even though a cycle of 200 was applied. The phase mapping indicates the dimensional selection of the 3D raw body is flexible according to the specific applications with ensured portability.

### Sensing Performance of 3D-FMS

With the successful formation of 3D-FMS, we herein characterized the sensing performance of the device from induced voltage signals. First, we simulated the magnetic field distribution surrounding the 3D-FMS as demonstrated in Figure S14. The results show that the edge of the device exhibits an intensified magnetic strength, which plays an important role in causing magnetic flux variation during the stretching and oscillating process. By placing the magnetic probe at the bottom of the 3D-FMS (Figure 3a), we monitored the real-time change of magnetic field intensity at the bottom of a typical 3D-FMS (dimension of 3 × 3 × 3.5 mm) by a 0.5 mm stepwise stretching. As shown in Figure 3b, the variation of the magnetic field intensity in the *Z*-axis is consistent with the stretch and release of the device in axial displacement (see Figure S15 for the variation of magnetic field intensity in the *X* and *Y* directions). The result reveals a characteristic growth pattern in the *Z*-axis magnetic field intensity on the bottom surface of the 3D-FMS. As the stretching distance extends from 0 to 3 mm, the magnetic field intensity demonstrates a nonlinear enhancement from approximately 0.1 mT to 1.5 mT, exhibiting progressive saturation behavior with increased deformation. In the subsequent release stage, the magnitude of the magnetic field intensity is continuously decreased with reduced stretching length. The stability and reversibility of localized magnetic flux are essential for electromotive force generation to serve as a platform for surface adhesion perception.

**Figure 3:**
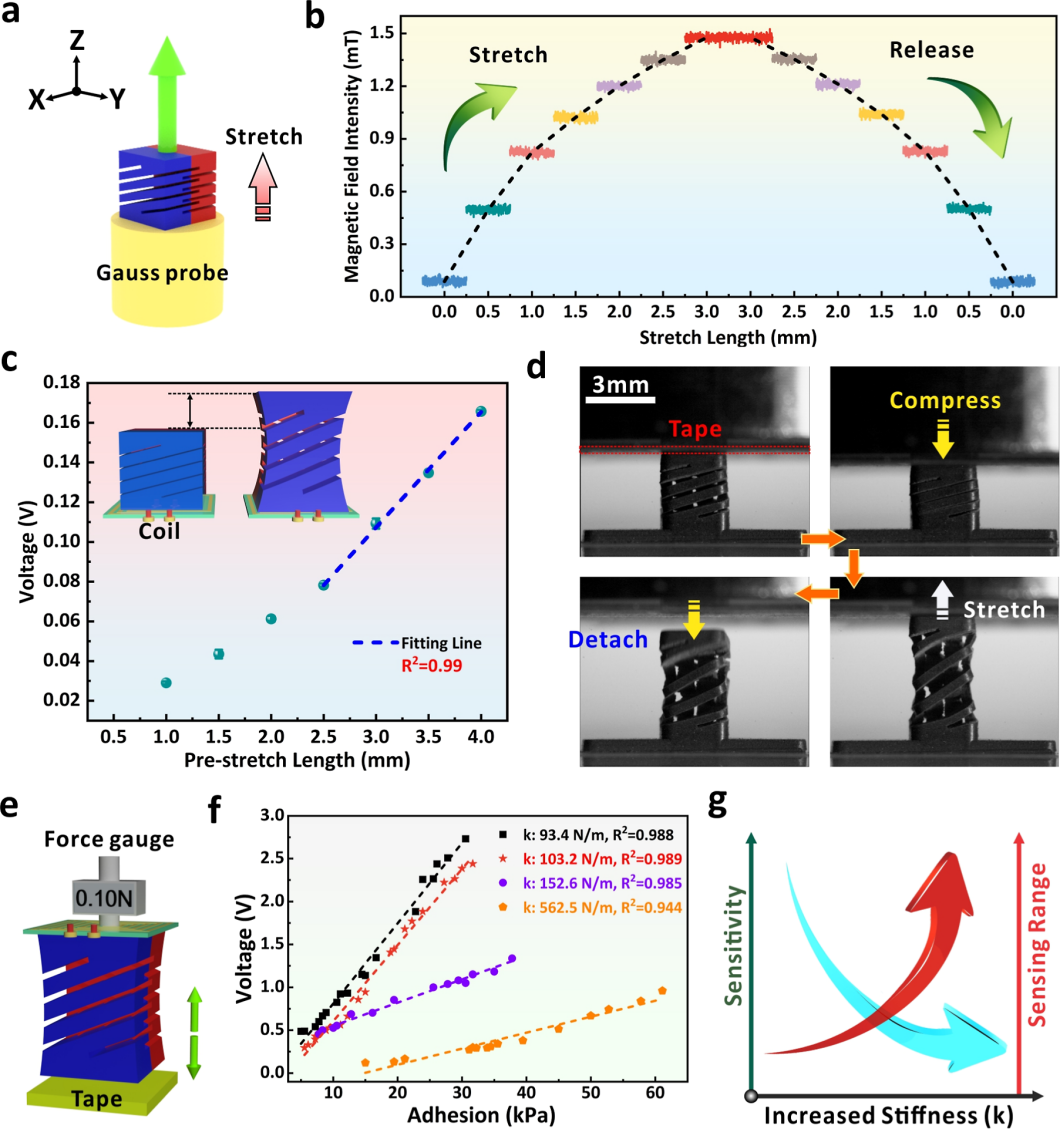
(a) Schematic diagram of the experimental setup for magnetic field intensity measurement. The green arrow shows the stretching direction. (b) Magnetic field intensity variations in the *Z*-axis when the 3D-FMS is stretched and released at specific length. (c) Dependence of peak voltage values on the prestretch length of the device. (d) Real-time record of the mechanical deformation when the 3D-FMS was exposed to adhesive tape. The scale bar is 3 mm for all optical images. (e) Schematic diagram of the experimental setup for adhesion measurement of four 3D-FMS with different stiffness. (f) Relationship between peak voltage and adhesion strength from different devices. (g) Summary of the effect of device stiffness on the sensitivity and the sensing range.

Before applying the device for quantitative adhesion perception, we evaluated the relationship between the stretching length and the induced voltage. In principle, the stretching length is one critical parameter that affects the variation of magnetic flux at the instant of device–surface separation. Herein, we used a clamp to prestretch the 3D-FMS to a specific displacement, followed by a quick release to record the voltage at that moment. With a prestretched displacement of 0.5–3 mm, the instant release of 3D-FMS results in a sudden magnetic flux in the coil to induce electrical voltage as presented in Figure 3c. With increased prestretch length, the generated voltage is accordingly enhanced because of a more significant change in magnetic flux. Significantly, the induced electromotive force exhibits a linear relationship (*R* ^2^ = 0.99) with the prestretch length of 3D-FMS. Figure 3d further provides the complete touch–compress–stretch–detach process by a high-speed camera (Video S1). When the adhesive tape was pressed toward the device with predefined maximum pressure, it would first compress the device to a corresponding displacement. After that, the withdrawal of the tape would stretch the 3D-FMS in an axial orientation because of the adhesive strength. At the moment when the inherent elastic force is comparable with the adhesion, the top layer of 3D-FMS would detach from the surface of the tape. This instant detachment then contributes to the generation of electromotive force to electrically reflect the adhesive properties.

To systematically investigate the sensing performance based on the geometrical property, we fabricated a series of 3D-FMS with different stiffness values for evaluation. Herein, the cutting cycles of four devices were set as 40, 60, 80, and 100, while other parameters remain unchanged (mass of NdFeB:Ecoflex:PDMS as 2:1:1, θ of 15°, and *S* of 0.5 mm). These 3D-FMS underwent a programmed compression–detachment cycle until complete interfacial separation from the same adhesive tape. As shown in Figure 3e, the force gauge was attached onto the coil layer, and the assembly was moved toward the adhesive tape for adhesion perception. Through pressing the 3D-FMS on an adhesive surface with different pressure, the adhesion was calculated by dividing the maximum force displayed on the force gauge during the separation process, by the surface area (3 mm × 3 mm) of the device. Once separated, the device quickly released and oscillated around the equilibrium position to output the induced voltage as the sensing signal. The maximum value of induced voltage upon separation was applied as the sensing output, which was indicated as the *Y*-axis in the plot (Figure 3f). The graphs present the dependence of voltage magnitude based on different adhesive performance that is associated with the device stiffness and applied pressure during approaching. With increasing 3D-FMS stiffness, the output voltage signal gradually decreased under identical adhesion conditions. The 3D-FMS with lower stiffness exhibited linear sensing with a sensitivity of 0.094 V·kPa^–1^ within a range of 5–30 kPa, whereas the device with a higher stiffness value exhibited a broader detection range (15–60 kPa) and reduced sensitivity (0.018 V·kPa^–1^). Figure 3g summarizes the relationship among the stiffness of 3D-FMS, sensitivity, and detection range. In principle, further increased stiffness would reduce the sensing sensitivity, while the detection range of adhesion can conversely be expanded because of the intensified mechanical strength. Alternatively, the sensor with further decreased stiffness can possibly improve the sensitivity, while the working range would be narrow due to the mechanical damage that might occur during the sensing process. From this perspective, such behavior allows us to customize the performance of 3D-FMS toward specific applications through the parameter regulation.

### Reliability and Demonstration

As an adhesion sensor, 3D-FMS should demonstrate reliability across diverse operating conditions. First, we investigated the signal deviation when the 3D-FMS approaches and leaves the target tape with different speeds based on the experimental setup described above (Figure 3e). As shown in Figure 4a, under constant maximum pressure, slower movement (1.1 mm/min) produced higher voltage outputs due to the prolonged contact duration between the device and tape. The extended interaction allowed stronger interfacial bonding that finally results in more significant structural elongation before complete detachment (region I). With a higher motion speed, the contact time would inevitably be reduced that leads to a lower sensed voltage. Stable voltage signals were maintained within the motion speed range of 25.1–60.1 mm/min (region II), indicating that the sensed adhesion is independent of the approaching speed under such an investigated range. As presented in the plot, insignificant signal variation was observed for a broad motion speed, which indicates that the surface adhesive property can be precisely reflected if a maximum pressure was maintained. Note that the sensed voltage increases when the platform motion speed is further increased (region III), which is attributed to the increased maximum pressure under this speed range (Figure S16). The relationship between peak force and the motion speed shows that a higher speed would normally result in a larger peak compression force during the contact. Consequently, an intensified contact would result in the trend of voltage increase, as circled in the plot (Figure 4b). The consistence between actual and sensed values also convinces the reliability of the 3D-FMS as a biomimetic tool for surface adhesion perception. Here, the actual adhesion value was obtained by dividing the maximum stretching force recorded from the force gauge by the surface area (3 mm × 3 mm) of the device. The sensed adhesion value is equal to 11.278 × voltage + 3.174, after the calibration of the device under study.

**Figure 4:**
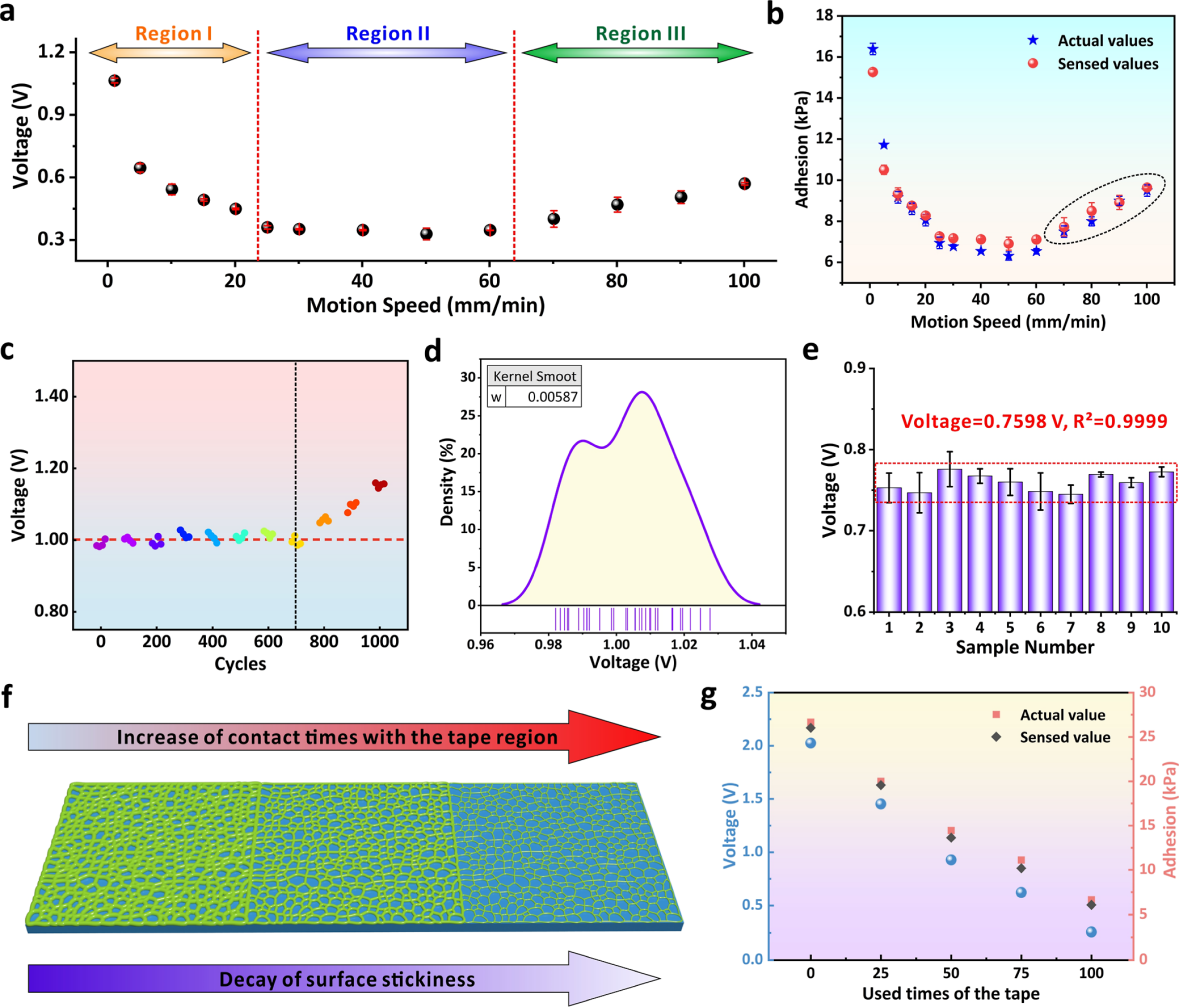
(a) Induced voltage values when different motion speeds were applied on the 3D-FMS to move toward the adhesive tape for measurement. (b) Comparison of the actual and sensed adhesion values across different motion speeds. (c) Evaluation of long-term stability of the 3D-FMS via periodical deformation up to 1000 cycles. (d) Distribution density of the induced voltage across different sensing cycles. (e) Peak voltages obtained from different samples prepared by the same templates and fabrication parameters during the laser processing. All tests were processed with the same type of adhesive tape and the maximum pressure. (f) Schematic diagram of the regional tape with different adhesion after cyclic uses. (g) Comparison of the actual and sensed voltage signals when the device was applied to the tape that has been used for different cycles.

Long-term stability is another critical parameter to elevate the practicality of 3D-FMS as a wearable adhesion sensor. Figure 4c shows the induced voltage values when periodical stretching–release operations were applied to the 3D-FMS toward the same adhesive surface. In this measurement, we changed the tape every 100 cycles to ensure that the surface stickiness of the tape was not obviously changed after periodical uses. It can be observed that within 700 cycles, the recorded voltage signal of the 3D-FMS remains almost consistent as the initial value. The distribution density of voltage values in Figure 4d further confirms the consistence of the sensed values across different cycles. The results experimentally convince the robustness of the flexible matrix, which is able to support periodical stretching so that the difference of induced voltages is negligible even after multiple uses. When the cyclic number increases afterward, the measured voltage exhibited a slight rise with a drift rate of ∼16% after 1,000 cycles. The observed deviation here primarily arises from progressive tearing of the cut lines during repeated cycling, which inevitably increases the average diameter of the spring, thereby reducing the stiffness of the 3D-FMS. As mentioned above, stiffness reduction would lead to higher sensed voltage signals for the same adhesive surface due to the weaker mechanical resistance from the intrinsic device matrix. Furthermore, the template-based fabrication method enables batch production of the raw bodies at a low cost, allowing straightforward replacement with fresh 3D-FMS devices when cyclic application times exceed the threshold. For this purpose, we also investigated the voltage repeatability among different 3D-FMS devices that were fabricated by the same parameters, e.g., mass compositions and cutting cycle, angle, and space. Each sample was pressed against different positions of the same tape four times under identical motion speeds and maximum pressure, and the average sensed voltage signal was recorded as presented in Figure 4e. The sensed voltage values of all sensors stabilized at approximately 0.7598 V (*R* ^2^ = 0.9999), demonstrating good consistency among different devices thanks to the reproducible methodology. Here, $R^{2}=1-\frac{\Sigma {\left(y-ŷ\right)}^{2}}{\Sigma {\left(y-\mathrm{y̅}\right)}^{2}}$ , where *y* is the true voltage value of each measurement, *ŷ* is the predicted value of the model, and *y̅* is the average of the true values. The results indicate the reliability of the wearable device toward real applications for biomimetic perception of surface stickiness.

As multiple contacts with an adhesive tape might cause a decrease of adhesive strength, we also used 3D-FMS to evaluate such changes in adhesion of a tape under different usage cycles. In principle, the 3D-FMS should be able to record the decay of adhesion via significant voltage drop, which is analogous to the human fingertip (Figure 4f). Considering this, we divided a fresh tape into five regions, and each region was contacted by fingers (with glove) to simulate the usage times of 0, 25, 50, 75, and 100 (see Figure S17 for the optical images of the regional tape). Subsequently, the device contacted different regions with the same maximum pressure, and the induced voltages were recorded at the moment of separation to reflect the respective surface adhesion. As shown in Figure 4g, the induced voltage signal from the 3D-FMS continuously decreased if the regional tape was contacted more times. Via converting the voltages to adhesion values, we compare the sensed values with actual reads from the force gauge (actual values). This is reasonable when the tape was applied in real applications for multiple cycles. Similar to the actual reading from a commercial gauge, the developed device could also provide accurate feedback on the surface adhesion. Furthermore, the sensed adhesion values closely match the actual measurements with an average error of ∼3.8%, indicating the reliability of the 3D-FMS in practical monitoring to reflect the adhesive decay of the tape. The results experimentally confirm that the developed sensor is capable to provide cyclic information on the adhesive tape from variation of the electrical signals. To further evaluate the sensing stability of 3D-FMS, we recorded the induced voltage when measurements were performed under different temperatures of the adhesive tape. Here, the tape was placed on a temperature-controlled stage, and the 3D-FMS was applied to the tape with different temperatures to induce the voltage for comparison. During the measurements, all other experimental conditions, e.g., initial pressure and motion speed, remain unchanged. As shown in Figure S18, the sensed voltage remains almost at the same level when the temperatures were increasing from 14 to 41 °C. This implies that the developed sensor is capable of sensing under a broad daily temperature range. Note that when the temperature of the tape reaches ∼55 °C, the significant surface melting would cause difficulty for the 3D-FMS to detach from the adhesive tape surface, which renders limitations to ensure reliable compressing and detaching for measurement. Furthermore, the effect of external magnetic fields on the 3D-FMS’s sensing ability is evaluated by placing a strong permanent magnet (surface magnetic field strength of ∼350 mT) at different distances from the bottom of the tape. As shown in Figure S19, the sensed adhesion value maintained at a relatively stable value when the adhesive tape was exposed to magnetic strengths up to ∼2.2 mT. By moving the permanent magnet toward the tape and the measurement system, the induced voltage was finally affected when the external magnetic field on the tape surface reaches ∼3 mT. This is attributed to the strong magnetic interaction between the magnetized sensor and surrounding magnetic field from the permanent magnet. However, the sensing results were almost unaffected across a broad external magnetic field, which is also much larger when compared with the natural magnetic field from the earth (typically in tens of microteslas, μT). From this perspective, the developed sensor is immune to the weak geomagnetic field from the earth, and thus, the reliability toward real applications can be well-ensured.

To evaluate the application potential of 3D-FMS, we established an interface to characterize its sensing capability in practical environments. As shown in Figure 5a, we assembled the coil layer and 3D-FMS onto a human finger, which was then electrically connected to the customized LabVIEW interface for wearable demonstrations. In the LabVIEW interface (Figure S20), the voltage curve obtained from each perception of surface adhesion is displayed in real time. The maximum voltage value was then extracted in the “*V*_max_” box. Through the built-in function, as discussed above, the corresponding linear equation was applied to directly convert “*V*_max_” to “Adhesion” for display. The target surfaces, nonadhesive (green dashed rectangle) or adhesive (red dashed rectangle), were firmly attached on a force gauge for sensing comparison. As a proof of concept, the fingertip with attached 3D-FMS was applied to press each region three times with gradually increasing force (Figure S21). Figure 5b depicts the difference when manual operation was applied to nonadhesive or adhesive regions. When applied onto the nonadhesive region, a separation between 3D-FMS and the surface would not cause obvious stretching of the device, resulting in weak electrical signals to reflect the nonadhesive property. Alternatively, the adhesive region would cause stretching of the device in the axial direction. Upon detachment, the 3D-FMS will oscillate along the motion direction, causing magnetic flux variation to induce significant electromotive force that is related to the operational parameters (e.g., force) and surface adhesion. The applied pressure values and corresponding sensed adhesion results are displayed in Figure 5c. The sensed adhesion value is equal to 18.058 × voltage – 2.378, after the calibration of the device under study. In the nonadhesive area, the sensed voltage signal from the 3D-FMS showed minimal changes with increased pressure, resulting in calculated adhesion all below 3 kPa even though a high force of 1.23 N was applied (Video S2). The video records the insignificant variation of adhesion values, which precisely reflects the real situation when the device was pressed toward a completely nonadhesive region. Note that the insignificant adhesion value from nonadhesive regions might be caused by the vibration of the 3D-FMS during the recession process. During the inherent vibration period, the variation of magnetic flux would thus induce electromotive forces within the coil layer. However, such a corresponding value is negligible when compared with the values from the adhesive region. For the adhesive area, the adhesion reached 5 kPa even under a gentle pressure of 0.36 N (Video S3). With the tape on top, the specific region clearly exhibits an adhesive property, as perceived by human fingertips. With increased pressing force up to 1.39 N, a tight interaction between the 3D-FMS and the adhesive tape results in an increased adhesion up to 35.5 kPa as demonstrated in the plot (Figure 5c). During the characterizations, the duration from the start of device rebound to the appearance of the adhesion value is ∼150 ms. With the built-in formula, the adhesion value can be simultaneously obtained as the voltage signals and displayed on the computer interface without an obvious delay. The demonstration exhibits the capability of 3D-FMS to perceive the adhesive property of a specific surface, which is attributed to the superiority of axial stretching/compression with induced electrical signals (electromotive force).

**Figure 5:**
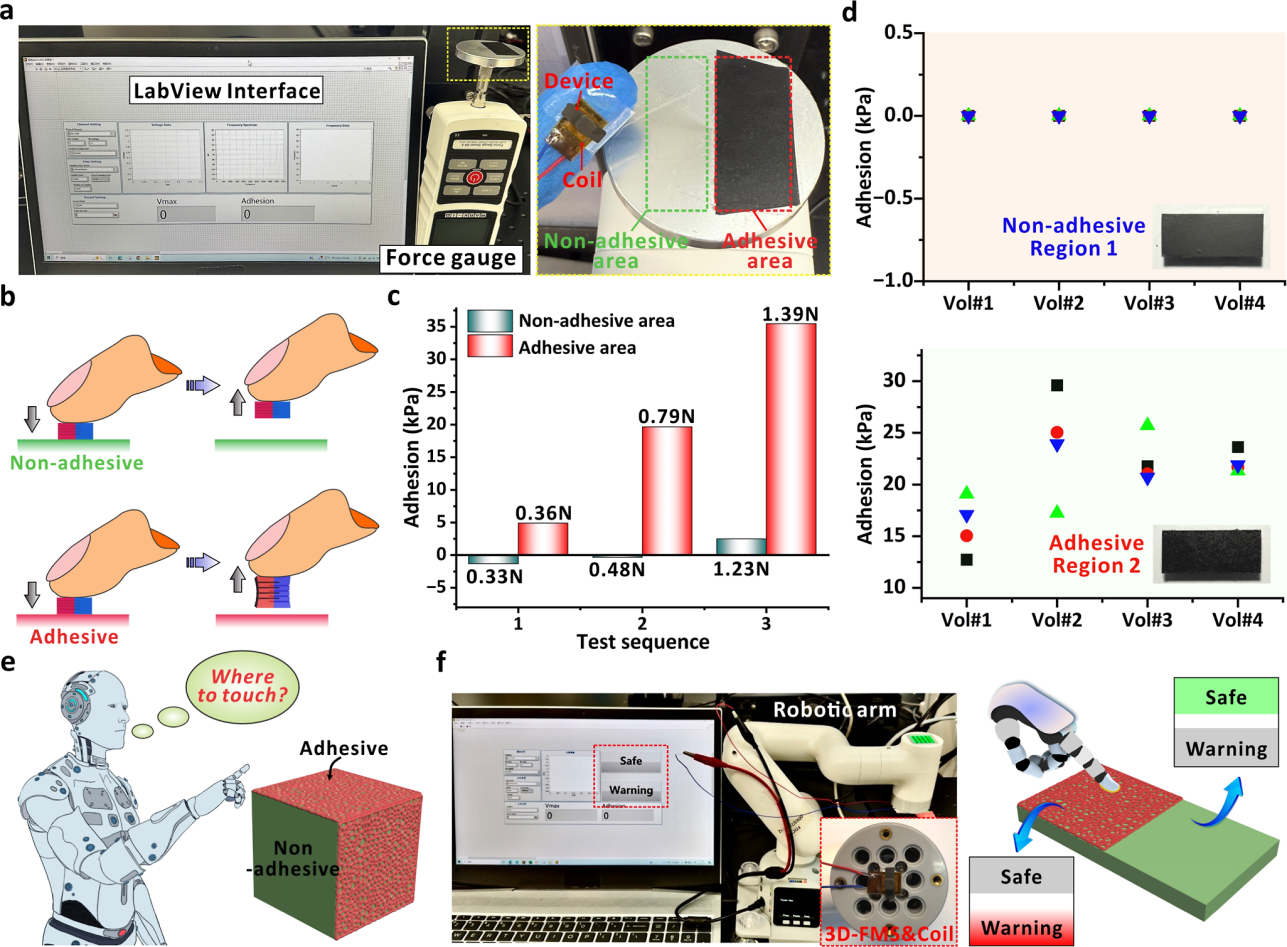
(a) Experimental setup for wearable demonstration, which is composed of a LabVIEW interface and the force gauge with a sensing platform. When touching the nonadhesive or adhesive regions, the 3D-FMS exhibits different behaviors to induce signal profiles for display. (b) Schematic diagram of the mechanical behaviors when 3D-FMS was pressed toward the nonadhesive and adhesive regions. (c) Relationship between the sensed adhesion and the applied force when the device was pressed against nonadhesive or adhesive area. (d) Results of sensed adhesion values from four volunteers when pressing the nonadhesive or adhesive regions. (e) Schematic diagram of surface adhesion perception for robots in daily operation. (f) Optical images of the experimental setup by attaching 3D-FMS onto a robotic arm to simulate the sensing process. When touching the adhesive region, a “warning” red light will be triggered, and the touch of the nonadhesive region would trigger the green “safe” light.

We further applied the sensing assembly to four volunteers for reliability evaluation (Video S4). To prevent visual recognition of surface adhesion before touch, we employed the same type of tape to ensure that the two regions maintained essentially identical morphological appearances (as inserted in Figure 5d). However, for region 1, we removed the adhesive layer on the tape using an alcohol-soaked cloth, whereas region 2 remained untreated. As a result, a significant difference in adhesion can be realized between these two regions. Subsequently, four volunteers were instructed to wear the 3D-FMS devices and conduct four arbitrary pressure applications in each region. The measured adhesive force magnitudes for both regions from each volunteer are presented in Figure 5d. The results demonstrate that for region 1 (nonadhesive), all measurements recorded an adhesion of ∼0 kPa, which aligns with the nonadhesive property as discussed above. When pressing on adhesive region 2, all measurements could render successful electrical signals due to the compression and stretching process of the 3D-FMS. Note that the variation in perceived adhesion values is mainly attributed to the applied pressure from volunteers. This is consistent with the daily operations in which perception of surface adhesion is also highly associated with the intensity of applied forces from humans. However, the signals clearly exhibit distinct behavior when compared with interaction of region 1, ensuring the capability to distinguish nonadhesive or adhesive surface properties by the wearable device.

As mentioned earlier, modern robots possess strong capabilities in grasping objects with diverse strategies. When handling objects with adhesive/nonadhesive surfaces, robots need to identify the regional surface property to ensure a safe contact for subsequent operation such as grasping or movement (Figure 5e). To address such a specific requirement, we assembled the setup as presented in Figure 5f. The LabVIEW interface (Figure S22) retains the functionality of extracting maximum voltages and converts them to adhesion in real time. For the common nonadhesive region, the sensed adhesion is within 3 kPa to trigger a green “Safe” light, indicating that the tested position is nonadhesive or weakly adhesive for safe contact. Conversely, the red “Warning” light will be activated if the sensed adhesion is high due to the stretching and release of the 3D-FMS. Video S5 demonstrates the entire demonstration process, from which we can observe that the device is capable of detecting the regional adhesion. As shown in Figure S23, five adhesive and nonadhesive regions were defined in sequence, and the device was continuously applied to touch the regions for adhesion perception. The activation sequence of the indicating lights matches the design of surface adhesion, showcasing 3D-FMS’s significant potential to automatically sense surface adhesion for applications in robotics. We expect that the study herein can provide an effective guidance for the design of wearable devices with the possibility of multifunctional explorations as human fingertips. For instance, through the coupling of a flexible resistive/capacitive sensing layer with integrated electrodes, the overall wearable system can simultaneously perceive the real-time tactile strength and surface adhesion during the interactions with a specific surface. Through combination of other tactile sensors with dedicated design, the 3D-FMS will also serve as a powerful tool to further explore the biomimetic sensing function of intelligent robots in the future.

## Conclusion

In summary, we reported a wearable 3D flexible magnetized spring that can mimic the function of human fingertips to perceive surface adhesive property. Via systematically investigating the mechanical behavior of the 3D-FMS, we established a spring-like model to study the influence from diverse parameters such as wire diameter, spring diameter, and coil number. These parameters can be regulated based on the laser processing as well as the inherent elastic property of the flexible matrix. When the device was applied to an adhesive surface, the adhesion would lead to corresponding stretching in the axial direction. Upon complete separation, an electromotive force can be produced based on the law of electromagnetic induction at the instant of rebound. Experimental results have demonstrated that the peak voltage value is linearly related with the surface adhesion strength. Furthermore, optimization of the 3D-FMS has enabled the regulation of the sensing sensitivity and working range, providing the possibility to adapt to diverse application conditions. As demonstrated, the miniaturized device can be integrated into human finger or robotic arms to mimic the functionality of adhesion perception. Furthermore, we show that the sensed adhesion is comparable to the actual results which were obtained from the commercial force gauge. Along with the stability and robustness of the device, we anticipate that the 3D-FMS will provide a fresh approach to explore the applicable situations of flexible sensors, especially for cases where the interaction with nonadhesive/adhesive target surfaces is important.

## Methods

### Materials

The total wearable interface is composed of a 3D flexible magnetized spring (3D-FMS) and copper (Cu) coil layer. In particular, the materials for 3D-FMS include ferromagnetic materials and an elastic matrix. Neodymium–iron–boron (NdFeB) particles, purchased from Magnequench, China, were used to provide magnetic strength for induction of electromotive forces. Here, we applied NdFeB particles as a magnetic component because of the high remanent induction, large coercive field, and good thermal aging characteristics.^43–45^ With these features, the adopted NdFeB particles enable high-performance flexible sensors to be realized in a straightforward and reliable approach. The elastic materials, polydimethylsiloxane (PDMS) base, and curing agent (Sylgard 184 kit) were purchased from Dow Corning, USA, and the Ecoflex (model of 00-50) was obtained from Smooth-On, Inc., USA. The copper substrate used as the coil layer was purchased from Chengdu Do-itc New Material Co., Ltd., China. The standard tape used to simulate the adhesive surface was purchased from 3 M Company, USA.

### Preparation of the 3D-FMS

The raw body of the 3D-FMS can be simply obtained through a template method. To start with, a predesigned 3D shape cavity was formed on a poly­(methyl methacrylate) (PMMA) slab using an engraving machine (CNC-3020, JingYan Instruments & Technology Co., China). Then, PDMS gel (mass ratio of base to curing agent was 10:1), Ecoflex (mass ratio of part A to part B was 1:1), and NdFeB particles were weighed accurately with a specific mass ratio and mixed homogeneously. The mixture was then immediately degassed in a vacuum chamber to completely remove the air bubbles in the uncured gel. Subsequently, the mixture was injected into the cavity of the PMMA slab. After complete solidification in an oven at 80 °C (∼30 min), the cured PDMS/Ecoflex/NdFeB composite was peeled off from the PMMA slab, and the raw body was obtained.

Transforming the raw body into a 3D flexible spring requires a laser ablation process. A commercial laser engraving machine (LPKF ProtoLaser U4, LPKF Laser & Electronics AG, Germany) was used to sculpt two opposite faces of the raw body with a predesigned pattern. Here, the frequency, the power, and the mark speed of the laser were 50 kHz, 2 W, and 100 mm/s, respectively. The focus offset of the laser was equal to the length of the bottom face of the raw body. During the laser processing, we fixed the raw body in the system by commercial clamps to avoid sample movement. To change the cutting angle, the specific laser movement path and horizontal direction were preset in the processing software. The cutting space and depth can also be regulated through the software settings. In these cases, the sample maintained good stability when the laser spot moves along different angles on the raw body. After laser processing, the flexible spring was obtained with customized elasticity, which is essential to realize the surface perception as discussed before. Finally, the 3D flexible spring was exposed to an external magnetic field with a field intensity of ∼3 T and specific direction to conclude the preparation of 3D-FMS.

### Preparation of the Copper Coil Layer

The flexible coil was prepared by laser ablation with the same laser machine, as mentioned above. In this work, we fixed the overall dimension of the coil region to be comparable with a human fingertip while optimizing the parameters (e.g., loop number) for reliable signal generation. The width of each loop was 100 μm, and the distance between two adjacent conductive lines is also 100 μm. The coil consists of two layers (18 μm in thickness), which are separated by an insulating polyimide film (75 μm in thickness), and each layer contains 10 turns. The total electrical resistance of the coil sample is ∼6.2 Ω. After engraving, the coil was bathed in citric acid to remove the oxidized layer and a drop of conductive silver glue was deposited on the central hole to electrically connect the two separated layers. Finally, plastic capsulation was applied to the coil to avoid oxidation during the subsequent sensing demonstrations.

### Characterizations

Scanning electron microscopy (SEM, Sigma FE-SEM, Zeiss Corporation, Germany) equipped with an energy-dispersive spectrometer (EDS) was used to characterize the surface morphologies and element distributions of the 3D-FMS. Magnetic field distribution around the 3D-FMS was measured by a three-axis commercial Gauss-Tesla meter (DX-360, Dexin Mag, China). The stiffness of 3D-FMS was measured by a motorized platform (ESM303, Mark-10 Corporation, USA). An axial tensile force was applied to the 3D-FMS and the linear displacement of the 3D-FMS was recorded to reflect the stiffness as $k=\frac{F}{\delta }$ , where the applied force (*F*) was measured by the gauge and the displacement (δ) was recorded by the platform in real time. The current signals induced by 3D-FMS rebound and generated in the coil were amplified by a low noise current preamplifier (Model SR570, SRS, USA) and further captured by a multifunctional I/O device (USB-6341, National Instruments, USA). The real-time motion of the 3D-FMS was recorded by a high-speed camera (VEO 710S, Phantom, USA) with a rate of 10,000 frames per second. The wearable demonstrations were carried out by research volunteers with ensuring safety during the characterization. All data were analyzed and processed by the OriginLab software.

### Simulation of the Magnetic Field

COMSOL Multiphysics 6.1 was employed to simulate the magnetic field around the 3D-FMS under the steady state. The built-in geometry module was used to establish the 3D model of 3D-FMS. A cube with a side length of 3 mm was first constructed, and then ten rectangular thin slices with a slope of 15° and dimensions of 4 mm × 0.01 mm × 2.5 mm were constructed on the two opposite planes of the cube. The laser cutting result is simulated by Boolean difference set operation, where the cube subtracts the rectangular bodies to mimic the 3D-FMS. Sintered NdFeB was used as the material of the 3D-FMS with a magnetic field intensity of 1.0186*e* ^6^ A/m. The air atmosphere was set as a cube with a side length of 20 mm. “Magnetic field, no current” is selected as the physical field for the simulation model. The governing equations were ***H*** = −∇*V*_m_, ∇· ***B*** = 0, ***B*** = μ_0_μ_r_ ***H***, and ***B*** = μ_0_(***H*** + ***M***), where ***H*** is the magnetic field vector, *V*_m_ is the magnetic scalar potential, ***B*** is the magnetic flux density vector, μ_0_ is the permeability of vacuum, μ_r_ is the relative magnetic permeability, and ***M*** is the magnetization vector. The initial value of *V*_m_ was set as 0. The boundary conditions were set as ***n*** · ***B*** = 0.

### Configuration Parameters for the LabVIEW Interface

The built-in DAQmx series, subVIs, in LabVIEW were used to configure the acquisition parameters of the induced signals with a sampling rate of 10,000 Hz, sampling number of 1,000, and sampling trigger condition of >0.05 V (relative to baseline). Using the peak-seeking procedure and mathematical-operating procedure, peak voltage values and corresponding adhesion values can be extracted and calculated based on the established model.

## Supplementary Material













## Data Availability

Data supporting the findings of this study are available within the article (and its Supporting Information) and from the corresponding authors upon reasonable request.

## Notes

Schematic diagram of the fabrication process of 3D-FMS; scanning electron microscopy (SEM) and electron-dispersive spectroscopy (EDS) images of NdFeB particles, the raw body of the device, and the 3D-FMS; optical images of the copper-based coil layer; experimental setup for stiffness measurement of the 3D-FMS prepared by different laser processing parameters; optical images of the intersecting faces when different cut cycles were applied during the laser processing; optical images of the stretching behavior when the 3D-FMS was exposed to a force of 0.05 N; growth of cut depth with the increasing number of cut cycles; typical curves of stiffness measurement from devices prepared by different cut angles; optical images of face 1 when the devices were prepared by different cut spaces from 0.5 mm to 1.1 mm; schematic diagram of the cutting space between the adjacent laser trajectories; optical images of the 3D-FMS prepared by different cut angles; stiffness of the raw body prepared by different NdFeB and Ecoflex contents; optical images of the cutting and intersecting surfaces of the devices prepared by different compositions; simulation results of the magnetic field distribution around the 3D-FMS; magnetic field intensities in the *X*-axis and *Y*-axis when the 3D-FMS is stretched to different lengths; force spectra when the 3D-FMS was exposed to adhesive tape by different motion speeds with an enlarged view; optical image of the tape for evaluation of device performance if exposed to the tape region that has been used for different times; sensing performance of the 3D-FMS under different temperatures of the same adhesive tape; sensed adhesion values when the 3D-FMS was applied to the tape under different intensities of the external magnetic field; LabVIEW script interface for wearable demonstration; optical images of the human fingertip pressing on nonadhesive and adhesive regions with attached 3D-FMS and coil layer; LabVIEW script interface for robotic demonstration; optical image of the robotic setup and demonstration results; detailed stiffness values for the 3D-FMS prepared by different cut parameters including the cycle, space, and angle; detailed stiffness values of the raw bodies for different raw material ratios; and detailed stiffness values of the 3D-FMS for different raw material ratios (PDF)
Record of the compress, stretch, and detach process of the 3D-FMS (MP4)
Sensing process of pressing at the nonadhesive area using the 3D-FMS (MP4)
Sensing process of pressing at the adhesive area using the 3D-FMS (MP4)
Volunteer test when the device was attached on a human fingertip to touch adhesive or nonadhesive regions (MP4)
Sensing process of the robot touching areas with different adhesion (MP4)
