# Supplementary material for: Bionic Perception of Surface Adhesion via a Magnetized Spring-like Sensor with Axial Stretchability
Source: ACS Nano. 2025 Jun 22;19(25):23465–78. doi: 10.1021/acsnano.5c07356 (PMC12224310; doi:10.1021/acsnano.5c07356)
Supplement: Supplementary file 1 [file nn5c07356_si_001.pdf]

Supplementary Information

Bionic Perception of Surface Adhesion via Magnetized Spring-like Sensor with Axial Stretchability

Yuanzhe Liang,[1] Biao Qi,[1] Ming Lei,[1] Yingyi Zhang,[1,2] Yifan Liu,[1] Yinning Zhou,[1] Jianyi Luo,[2] and Bingpu Zhou[1,3,]*

[1]Joint Key Laboratory of the Ministry of Education, Institute of Applied Physics and Materials Engineering, University of Macau, Avenida da Universidade, Taipa, Macau 999078, China

[2]Research Center of Flexible Sensing Materials and Devices, School of Applied Physics and Materials, Wuyi University, Jiangmen 529020, China

[3]Department of Physics and Chemistry, Faculty of Science and Technology, University of Macau, Avenida da Universidade, Taipa, Macau 999078, China

Corresponding Author

Bingpu Zhou, E-mail: bpzhou@um.edu.mo. Fax: +853-88222426. Tel: +853-88224196.

Keywords: *flexible tactile sensor, adhesion recognition, stickiness, 3D magnetized spring, laser processing*

Contents

Supplementary Figures. 2

Supplementary Tables. 25

Supplementary Videos. 28

Supplementary Figures.

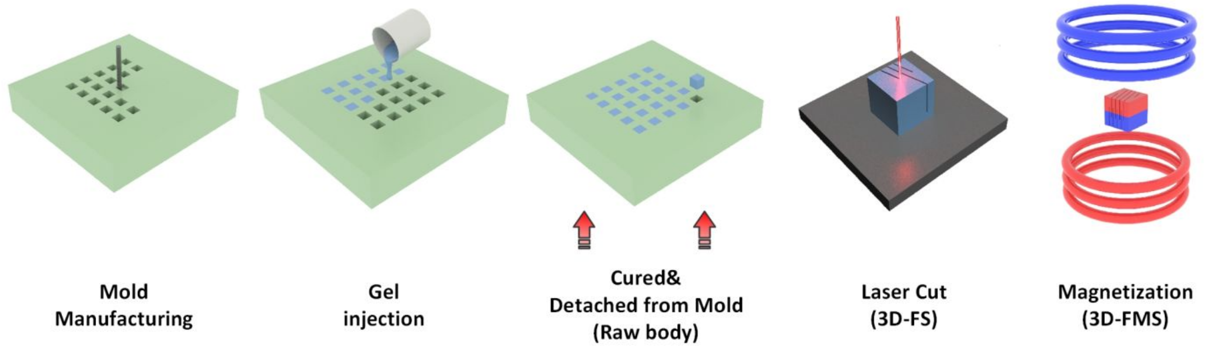

Figure S1. Schematic diagram of the whole fabrication process of the 3D-FMS.

Upon curing, the raw materials formed the raw body. Followed by a laser ablation process, the raw body was transformed into 3-dimensional flexible spring (3D-FS). And a 3-dimensional flexible magnetized spring (3D-FMS) was obtained after the magnetization process.

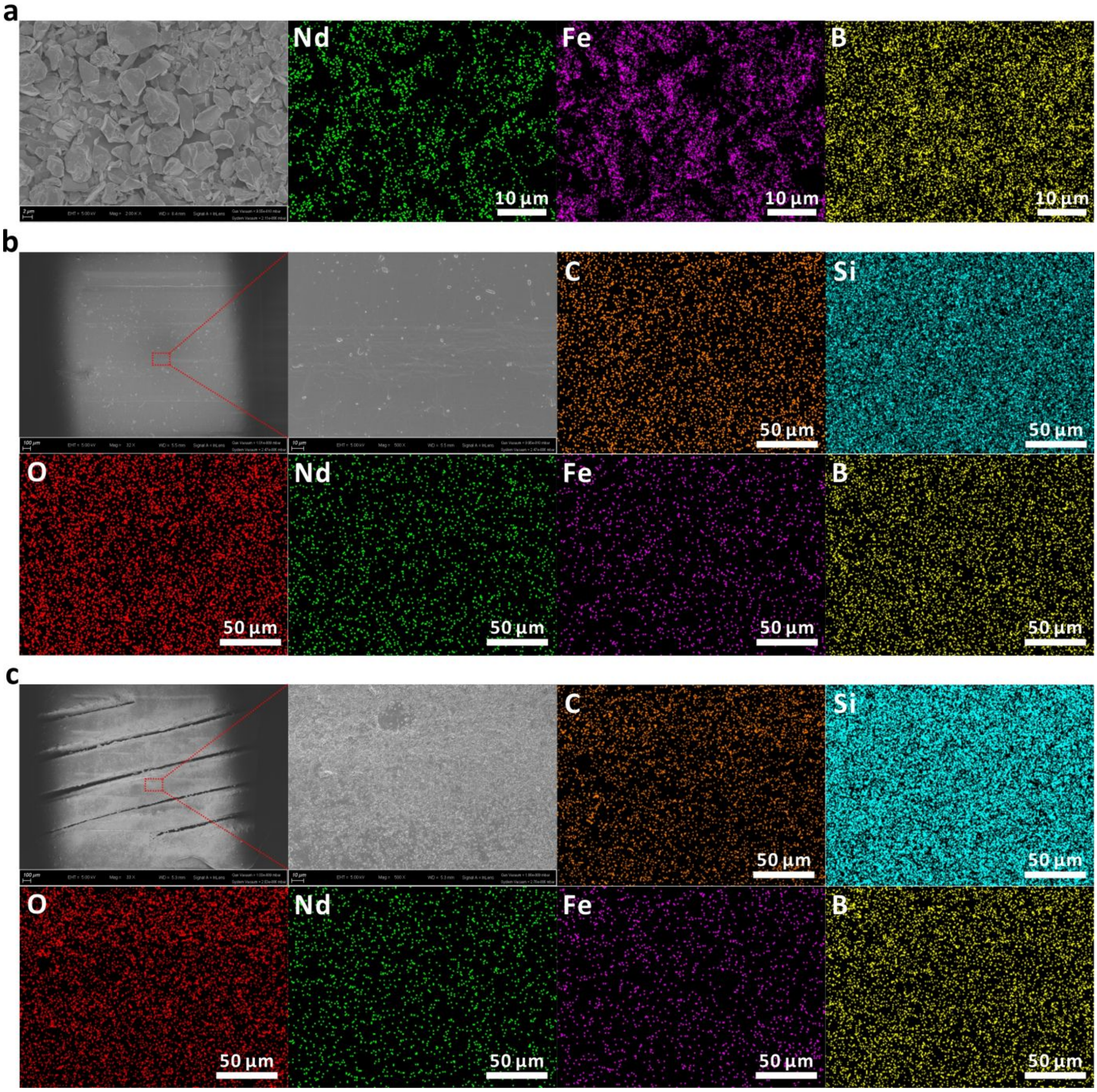

Figure S2. Scanning Electron Microscopy (SEM) and Electron Dispersive Spectroscopy (EDS) images of (a) NdFeB particles, (b) the raw body of the device and (c) the 3D-FMS.

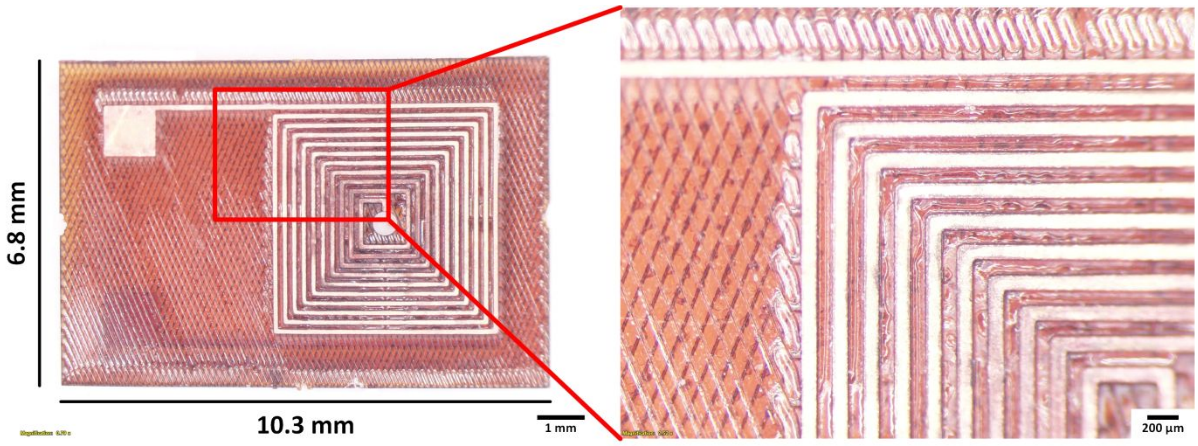

Figure S3. Optical images of the Copper-based coil layer.

The size of the coil is special customized, with a width of 6.8 mm and a length of 10.3 mm. The sensing area in the coil is slightly larger than the bottom surface of the 3D-FMS, approximately 5mm×5mm. The widths of each loop and the distance between two adjacent conductive lines are both 100 μm.

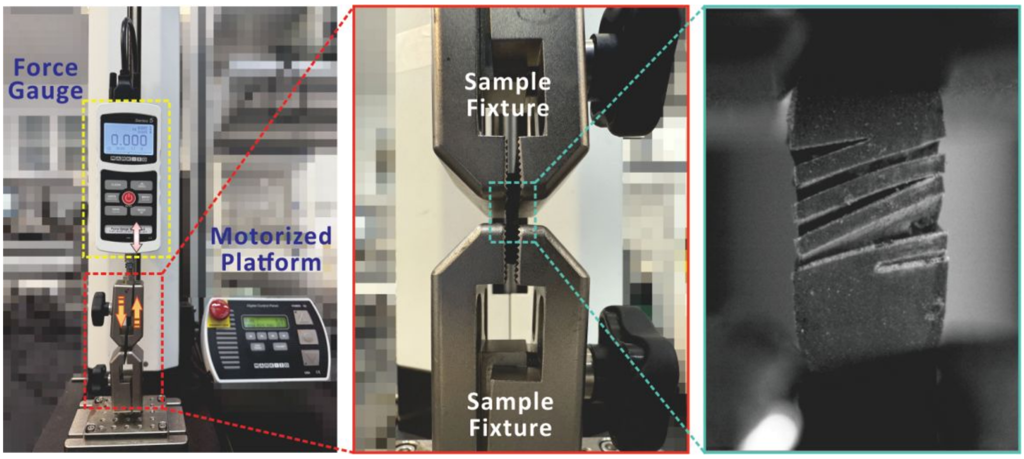

Figure S4. Experimental setup for stiffness measurement of the 3D-FMS prepared by different laser processing parameters.

In this experimental setup, the force gauge is applied to measure the force that is applied to the device. Simultaneously, the displacement of the 3D-FMS is recorded by the motorized platform. The optical image in the right panel also represents the device that is attached by the sample fixture.

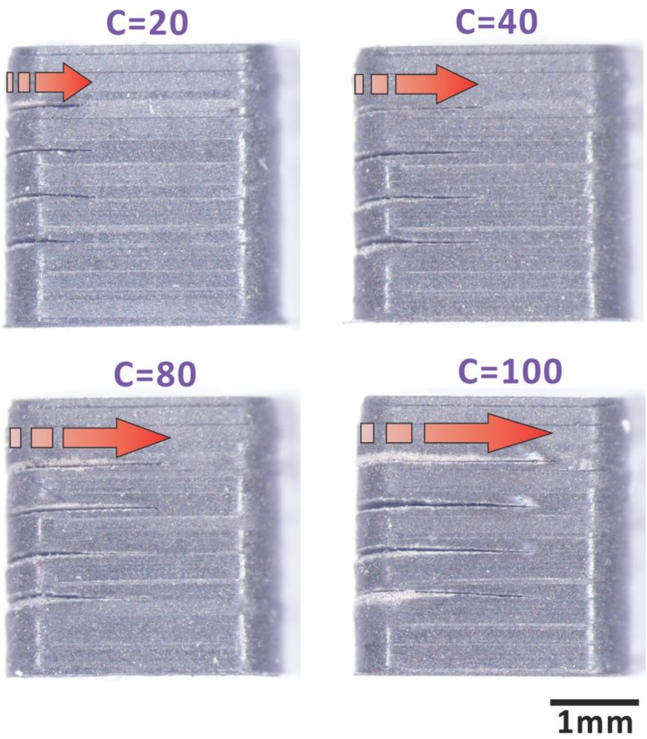

Figure S5. Optical images of the intersecting faces when different cut cycles were applied during the laser processing. "C" indicates the number of cycles.

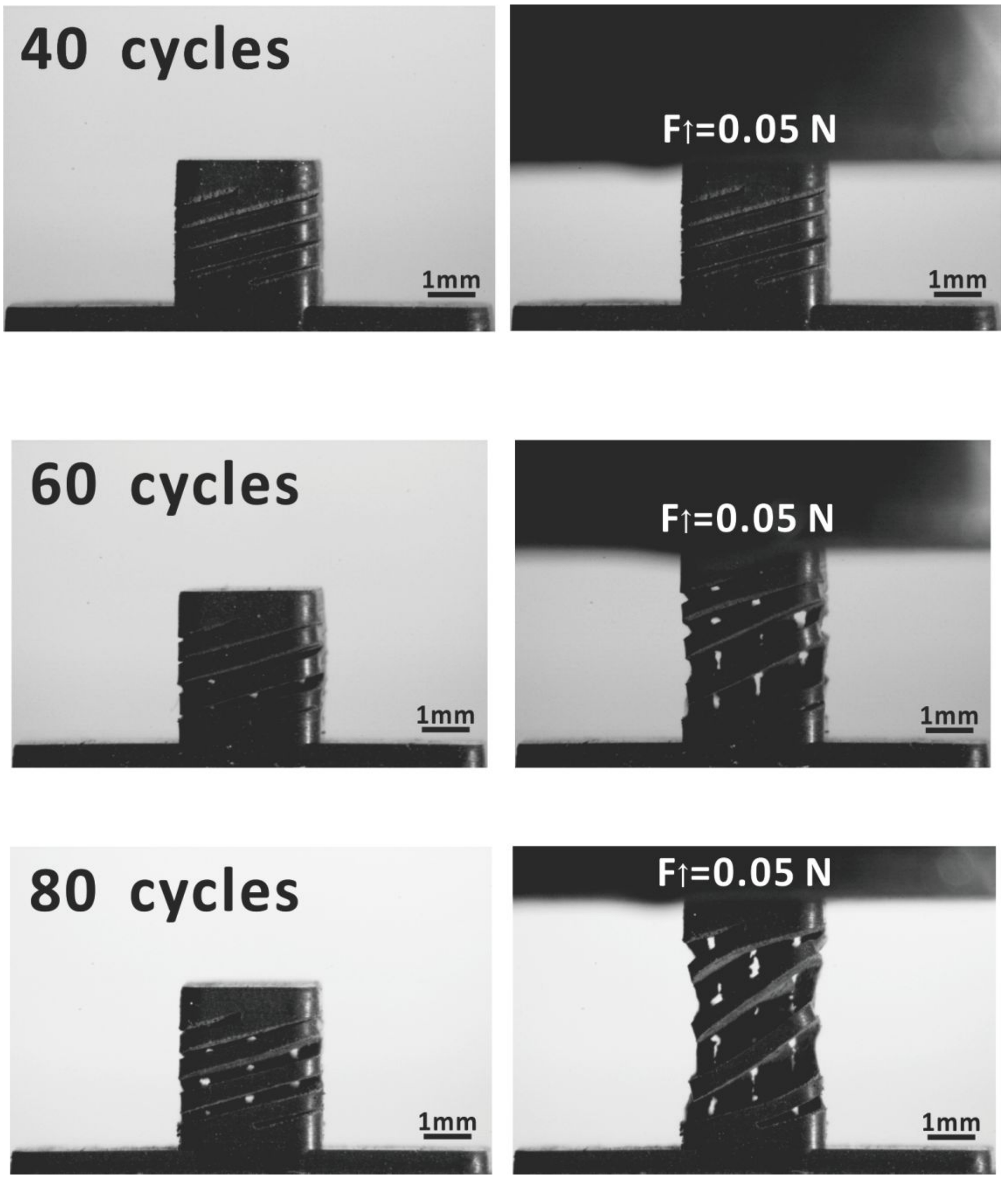

Figure S6. Optical images of the stretching behavior when the 3D-FMS was exposed to a force of 0.05 N. The devices were prepared by different cut cycles of 40, 60, and 80.

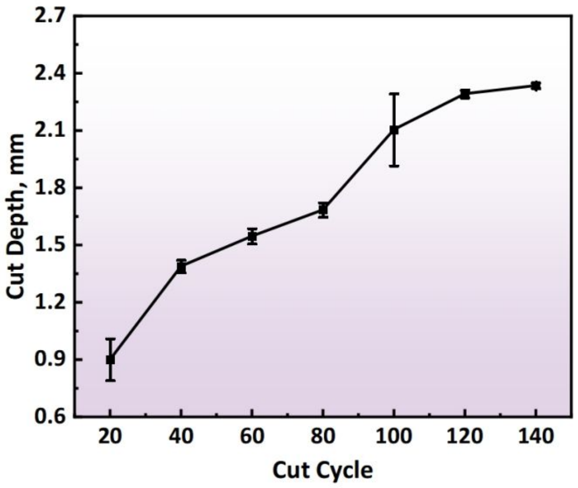

Figure S7. Growth of cut depth with the increasing number of cut cycle.

When the cut cycle is less than 120, the cut depth increases significantly with the cut cycle. Once the cut cycle is greater than 120, the cut depth remains relatively stable if the cut cycle is further increased.

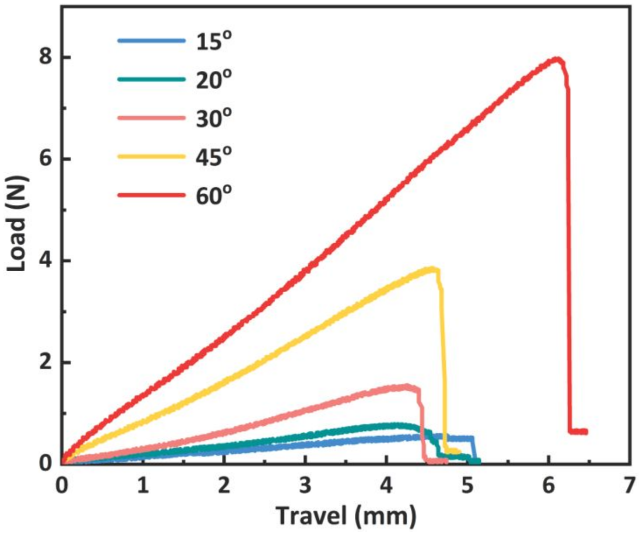

Figure S8. Typical curves of stiffness measurement from devices prepared by different cut angles.

As shown in the curves, the related load is continuously increased when the device was stretched with increased travel. The values of stiffness can then be obtained by calculating the ratio of applied load and the travel values. A larger stiffness is related with a larger cut angle as discussed in the main context.

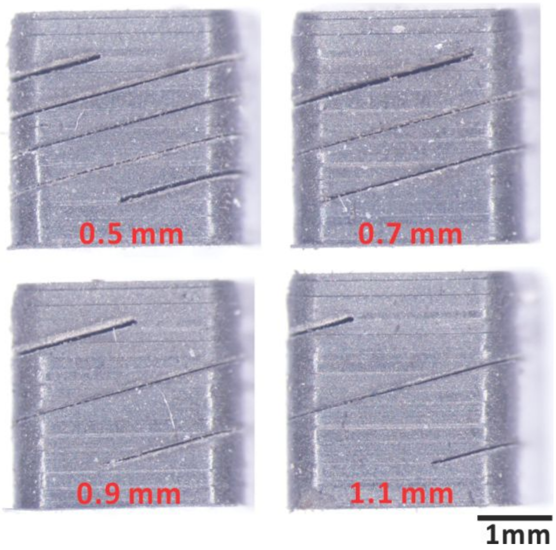

Figure S9. Optical images of Face 1 when the devices were prepared by different cut space from 0.5 mm to 1.1 mm.

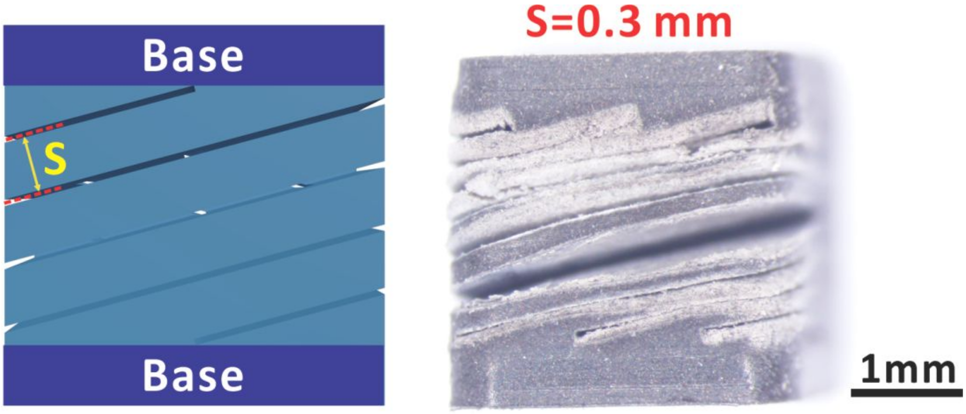

Figure S10. Schematic diagram of the cutting space between the adjacent laser trajectories.

The optical image shows the thermal effect on the device when the space (S) is small as 0.3 mm. If a narrow space is applied, the repeated laser processing within the region would bring obvious thermal effect as shown in the image.

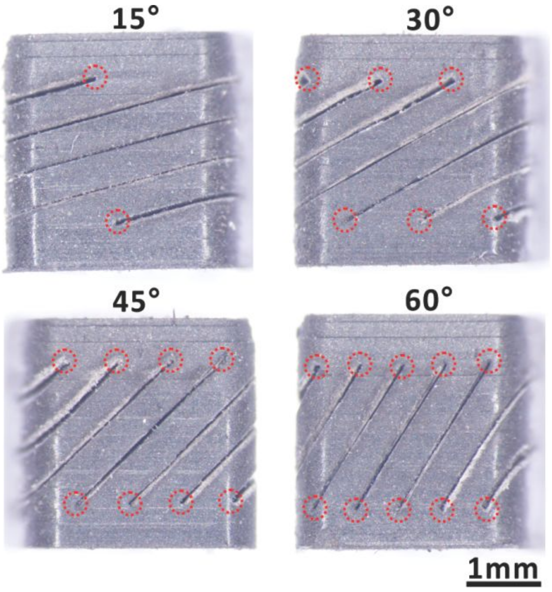

Figure S11. Optical images of the 3D-FMS prepared by different cut angles.

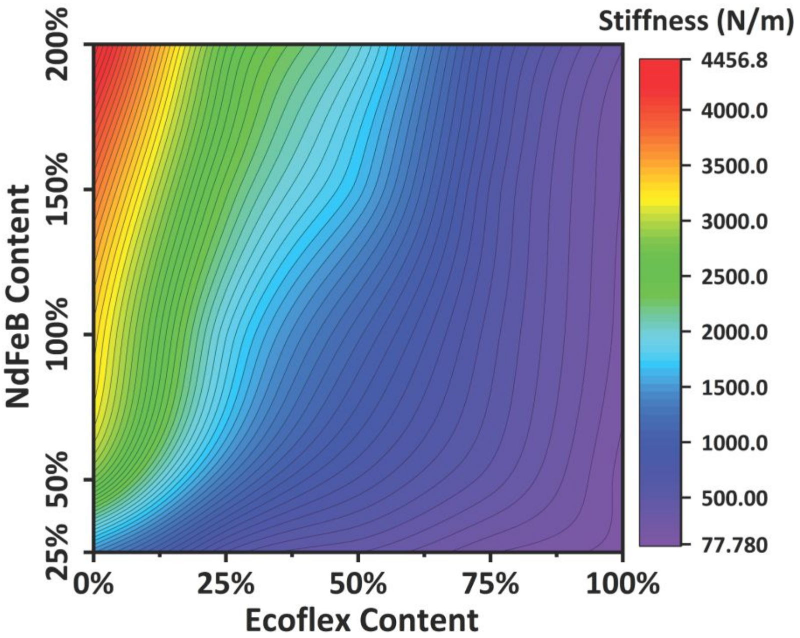

Figure S12. Stiffness of the raw body prepared by different NdFeB and Ecoflex contents.

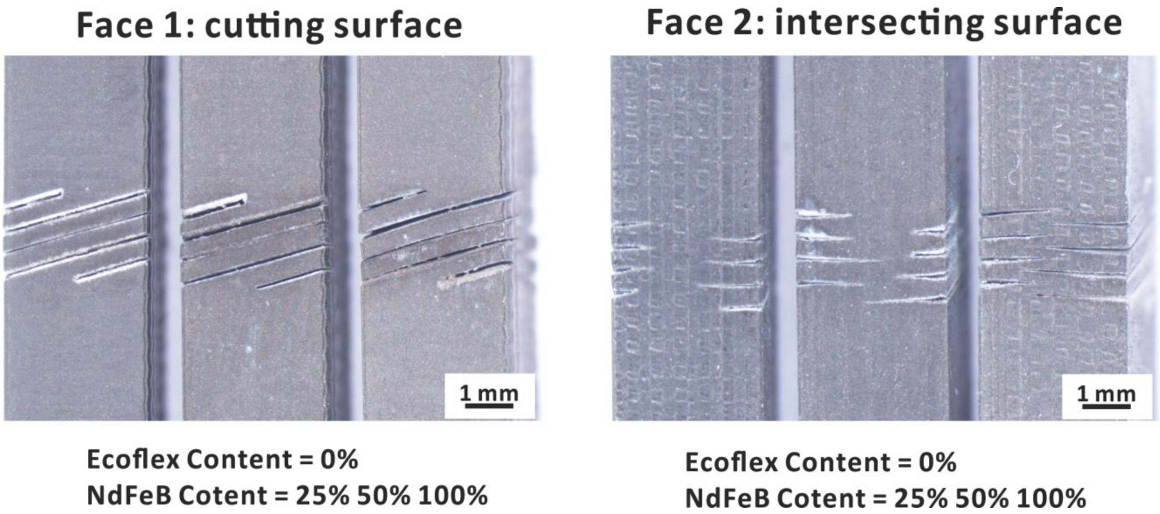

Figure S13. Optical images of the cutting and intersecting surfaces of the devices prepared by different compositions. All samples were prepared by PDMS matrix, and mixed with different NdFeB contents to evaluate the cut depth.

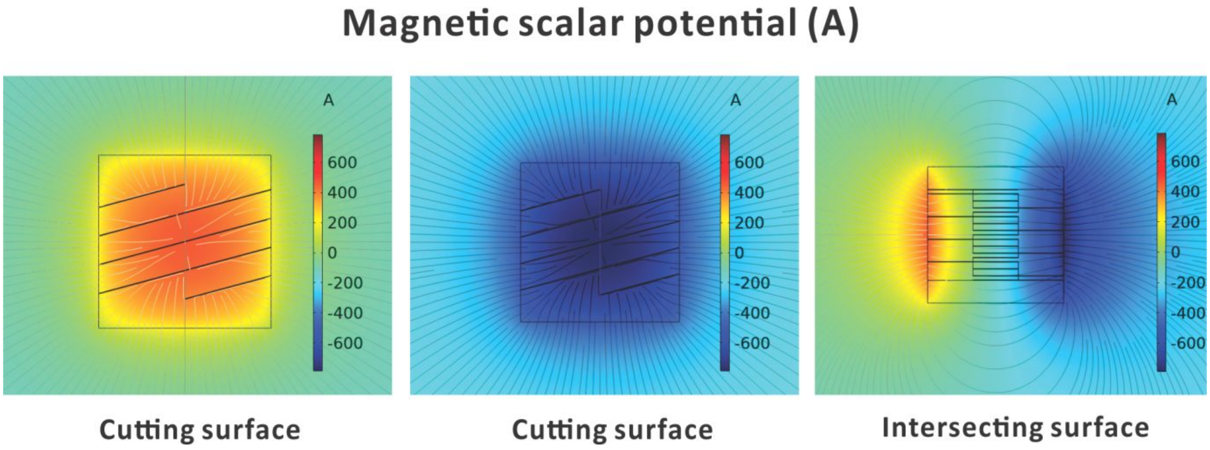

Figure S14. Simulation results of the magnetic field distribution around the 3D-FMS.

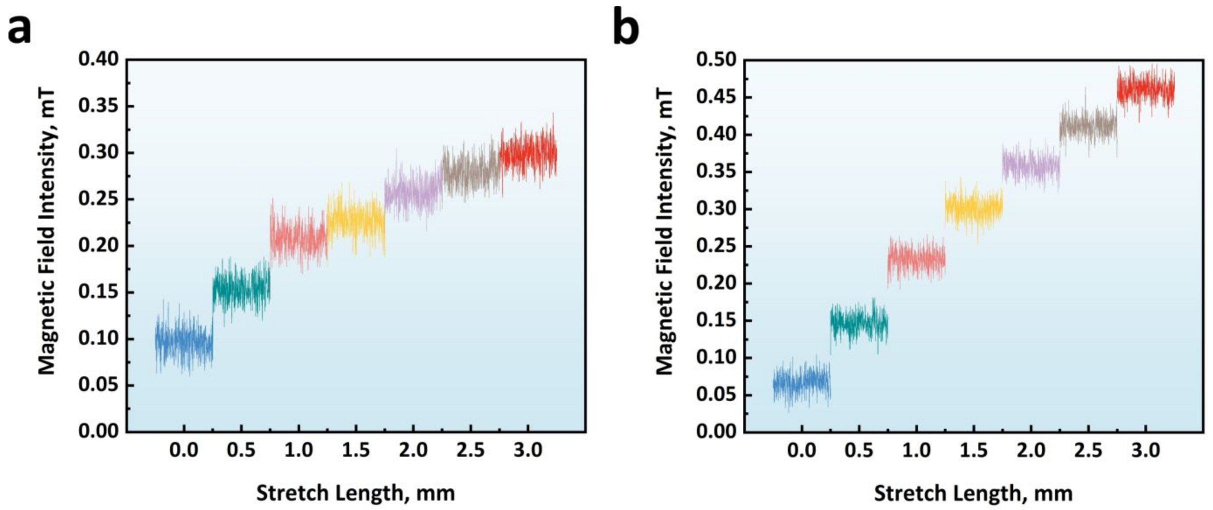

Figure S15. Magnetic field intensities in the (a) X-axis and (b) Y-axis when the 3D-FMS is stretched to different lengths.

The intensities in these axes were measured in real-time via a commercial Gauss-Tesla meter (DX-360, Dexin Mag, China).

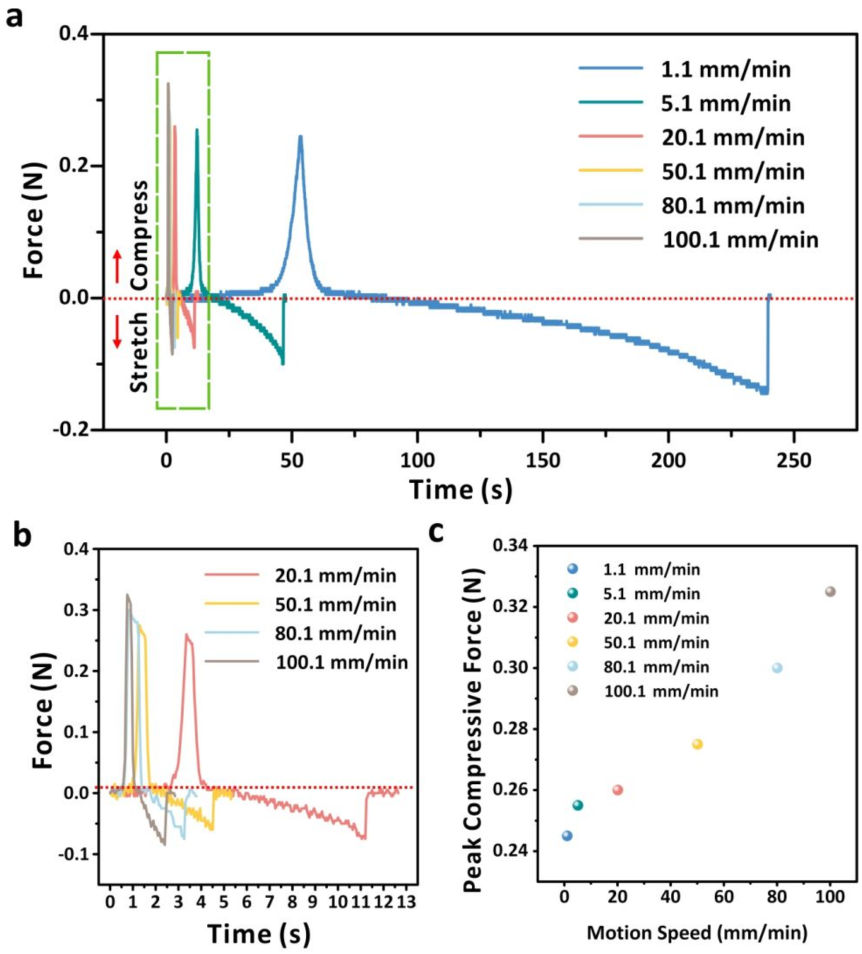

Figure S16. (a) Force spectra when the 3D-FMS was exposed to adhesive tape by different motion speeds, with enlarged view (b), and the peak force during compression (c).

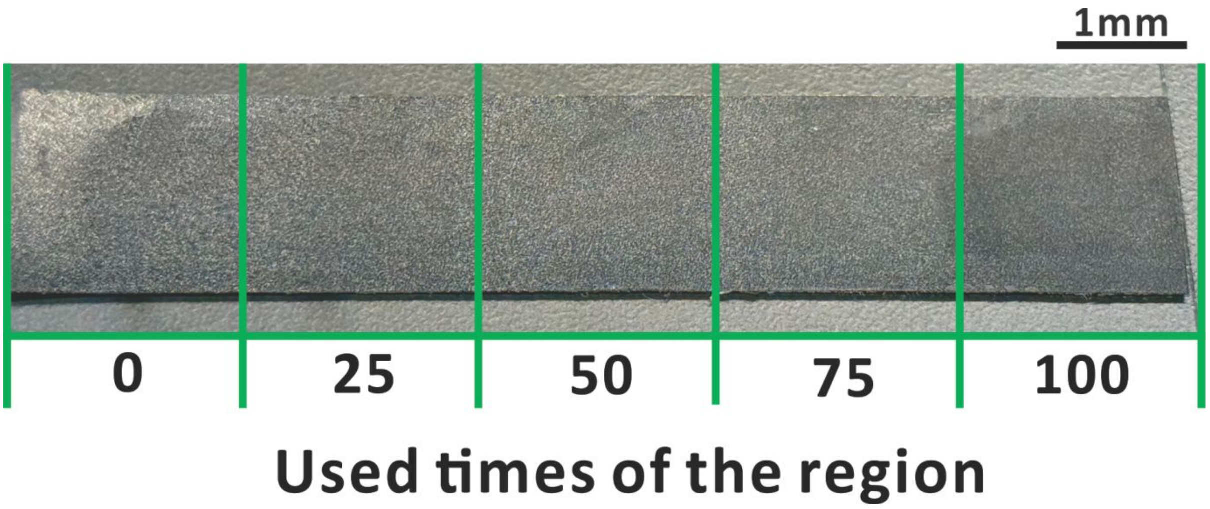

Figure S17. Optical image of the tape for evaluation of device performance if exposed to the tape region that has been used for different times.

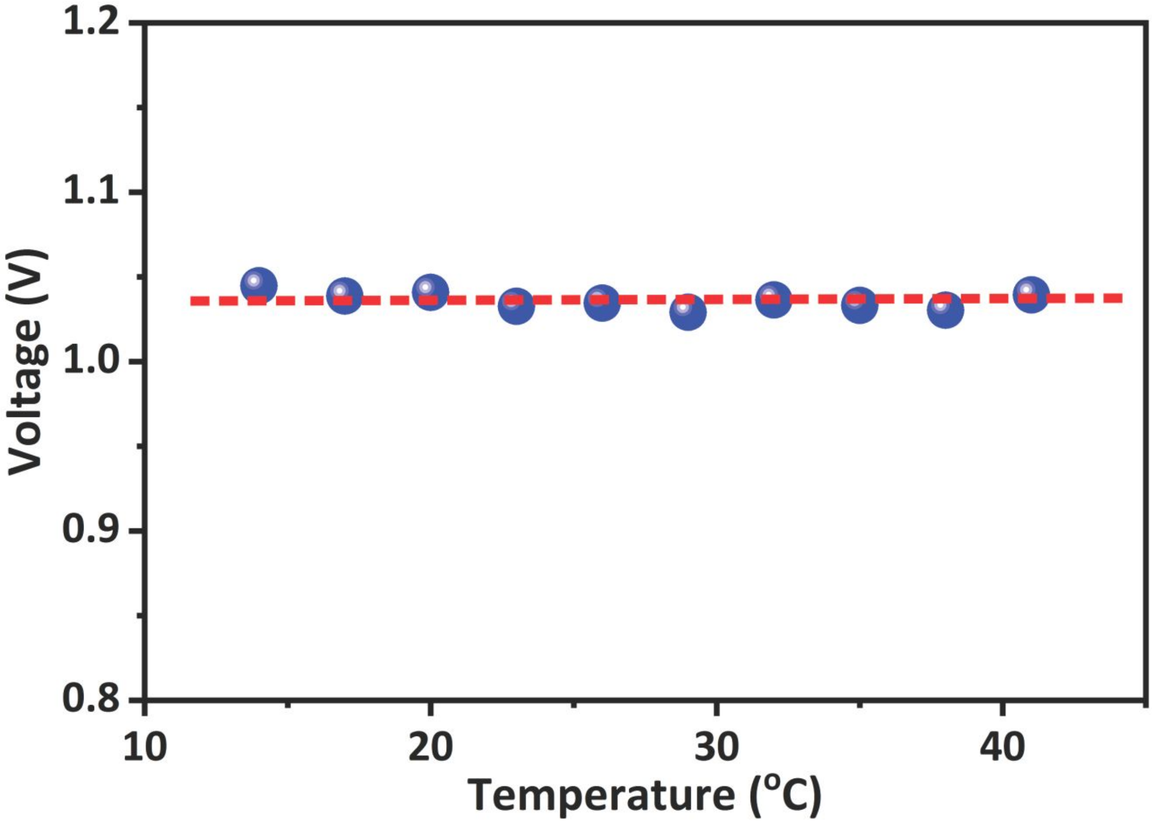

Figure S18. Sensing performance of the 3D-FMS under different temperatures of the same adhesive tape.

In the measurement, the tape was fixed on a temperature-controlled stage for surface temperature regulation. Consequently, the temperature effect can be monitored via pressing the sensor towards the adhesive tape, followed by a standard detaching process.

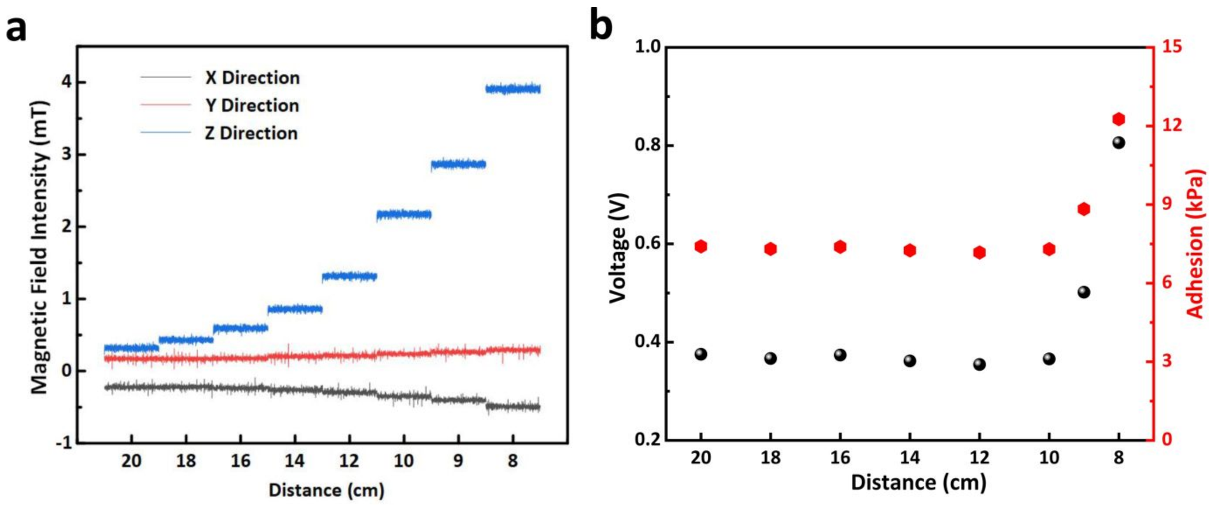

Figure S19. (a) Magnetic field intensity of the tape surface when a permanent magnet is placed at different distances from the tape. (b) Induced voltage signals and corresponding adhesion values based on different distances between the adhesive tape and the magnet.

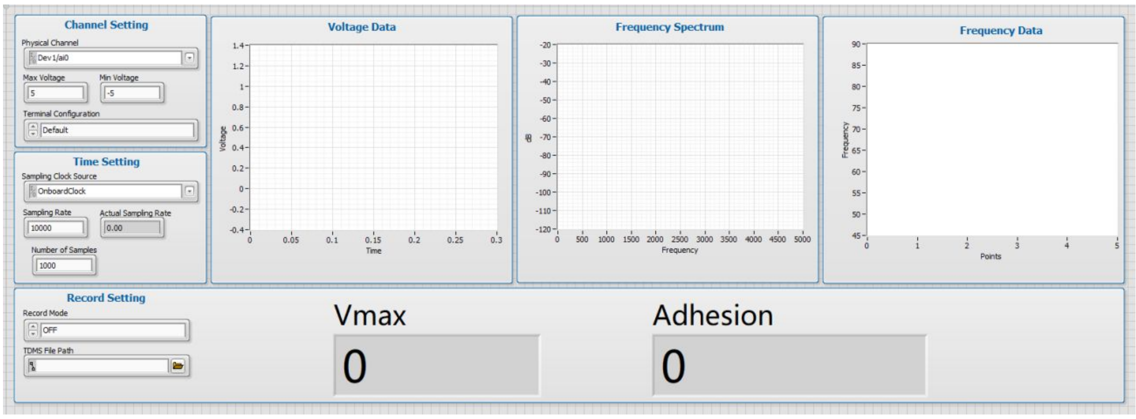

Figure S20. LabVIEW script interface for wearable demonstration.

The script is designed to record the induced voltage profiles during the rebound of the 3D-FMS. The peak voltage can be directly extracted and shown in the "Vmax" box in real time. And the adhesion value corresponding to the "Vmax" value is also calculated and displayed in the "Adhesion" box in real time.

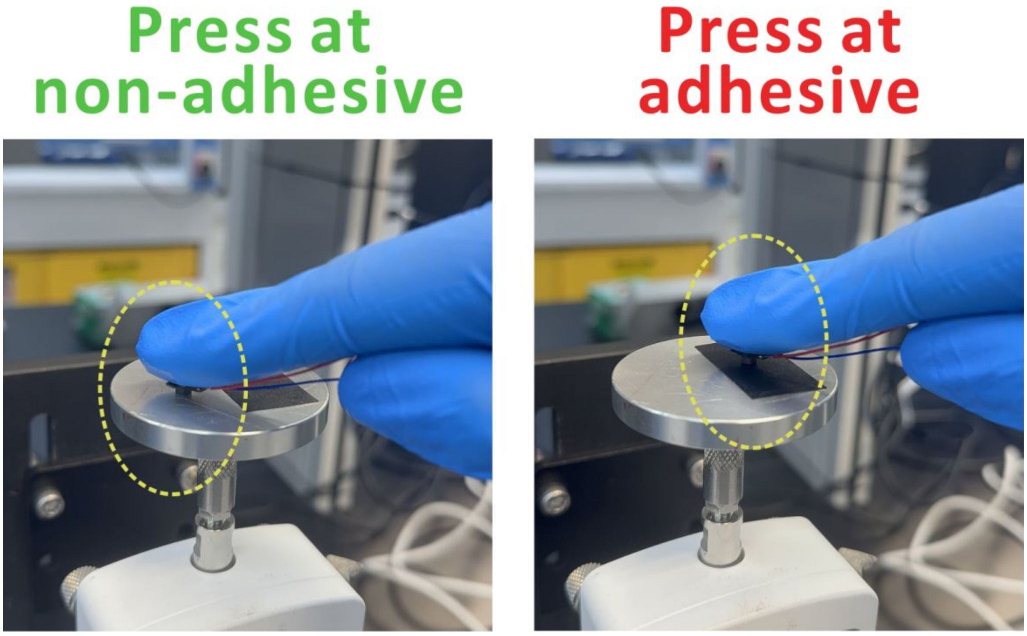

Figure S21. Optical images of the human fingertip pressing on non-adhesive and adhesive regions with attached 3D-FMS and coil layer.

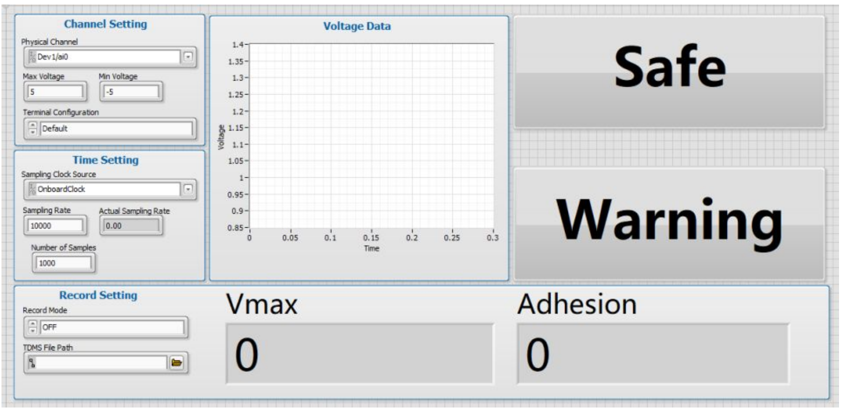

Figure S22. LabVIEW script interface for robotic demonstration.

The script also can display the voltage curve, peak voltage and corresponding adhesion during the rebound of the 3D-FMS in real time. In addition, two indicator lights are added to show whether it is safe for the robot to touch the test position.

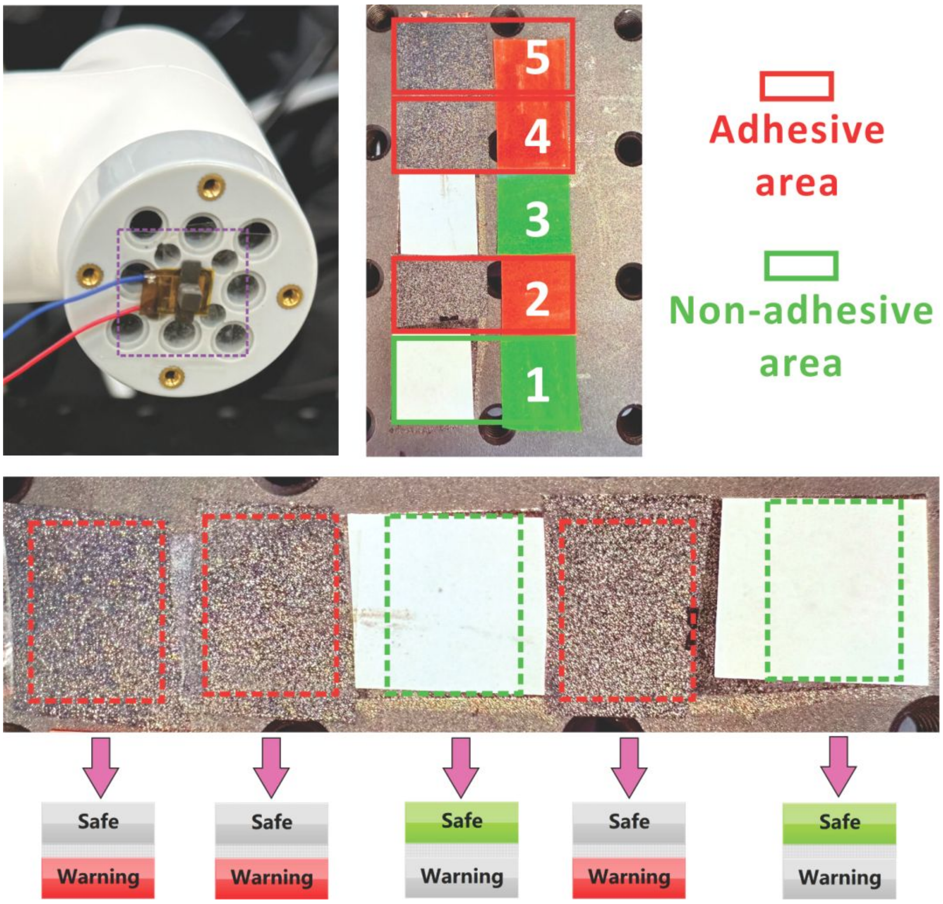

Figure S23. Optical image of the robotic setup and demonstration results.

Five regions were defined in sequence with non-adhesive, adhesive, non-adhesive, adhesive, and adhesive properties. Via touching specific locations, the green "Safe" and red "Warning" lights will be triggered according to the received voltage magnitudes.

Supplementary Tables.

Table S1. Detailed stiffness values for the 3D-FMS prepared by different cut parameters including the cycle, space, and angle.

Cut Cycle	Cut Space, mm	Cut Angle, degree	Stiffness k, N/m
40	0.5	15	562.54
60	0.5	15	152.62
80	0.5	15	103.16
100	0.5	15	93.36
120	0.5	15	83.63
140	0.5	15	83.14
80	0.3	15	90.84
80	0.7	15	224.25
80	0.9	15	340.13
80	1.1	15	453.29
120	0.3	15	64.05
120	0.7	15	158.11
120	0.9	15	243.88
120	1.1	15	301.62
120	0.5	10	38.25
120	0.5	20	137.86
120	0.5	30	296.59
120	0.5	45	645.72
120	0.5	60	970.56

Table S2. Detailed stiffness values of the raw bodies for different raw material ratios.

Ecoflex Content, wt.%	NdFeB Content, wt.%	Stiffness k, N/m
0	25	1469.83
25	25	613.51
50	25	411.76
75	25	207.46
100	25	77.78
0	50	2766.22
25	50	1321.60
50	50	750.65
75	50	448.18
100	50	100.22
0	100	3318.13
25	100	1883.65
50	100	1125.57
75	100	582.67
100	100	168.37
0	150	3760.11
25	150	2319.31
50	150	1631.04
75	150	677.79
100	150	192.29
0	200	4456.80
25	200	2660.75
50	200	1973.01
75	200	841.17
100	200	276.57

Table S3. Detailed stiffness values of the 3D-FMS for different raw material ratios. The laser parameters used to transform the raw body into 3D-FMS are the same for all samples.

Ecoflex Content, wt.%	NdFeB Content, wt.%	Stiffness k, N/m
0	25	1127.70
25	25	497.34
50	25	339.22
75	25	166.43
100	25	72.36
0	50	528.27
25	50	315.09
50	50	218.68
75	50	118.00
100	50	71.34
0	100	251.46
25	100	162.58
50	100	103.16
75	100	67.87
100	100	40.99
0	150	412.07
25	150	287.95
50	150	183.94
75	150	105.20
100	150	50.587
0	200	1171.13
25	200	319.22
50	200	238.94
75	200	129.06
100	200	100.46

Supplementary Videos.

Video S1. Record of the compress, stretch, and detach process of the 3D-FMS. The frame rate of the high-speed camera was 10000 fps (frames per second). The resulting video was 30 fps, so 1 s in the video should be about ~3 ms in real world when played at 1X speed.

Video S2. The sensing process of pressing at the non-adhesive area using the 3D-FMS. Without adhesive property, the device was easily to withdraw from the surface. No obvious electrical signals were received at the LabVIEW interface.

Video S3. The sensing process of pressing at the adhesive area using the 3D-FMS. With adhesive property, the device was stretched before withdrawing from the surface. Obvious electrical signals were received at the LabVIEW interface.

Video S4. Volunteer test when the device was attached on human fingertip to touch adhesive or non-adhesive regions. Four volunteers were informed of the test procedure after attaching the device on their index fingertip. The volunteers then pressed the adhesive tape or non-adhesive region to evaluate the potential for real application of the device. When pressing on adhesive tape, significant electrical signals were observed due to the stretching and vibration of the device. However, no signals were received when the device was pressed against the non-adhesive region.

Video S5. The sensing process of the robot touching areas with different adhesion. Whenever the robot touches an area, it will display the voltage sensing curve, maximum voltage value, and corresponding adhesion value for that specific region. Simultaneously, corresponding indicator lights will illuminate to show whether the area is safe to touch.
